# Preliminary Evaluation of Gemini-Surfactant-Based Formulations for Antifungal Seed Treatment in Wheat

**DOI:** 10.3390/molecules31101568

**Published:** 2026-05-08

**Authors:** Tomasz Szczygieł, Anna Koziróg, Anna Otlewska, Bogumił Brycki

**Affiliations:** 1 Institute of Fermentation Technology and Microbiology, Faculty of Biotechnology and Food Sciences, Lodz University of Technology, 90-530 Lodz, Poland; anna.otlewska@p.lodz.pl; 2 Interdisciplinary Doctoral School, Lodz University of Technology, 90-530 Lodz, Poland; 3 Department of Bioactive Products, Faculty of Chemistry, Adam Mickiewicz University Poznan, 61-614 Poznan, Poland; brycki@amu.edu.pl

**Keywords:** gemini surfactants, seed coating, *Fusarium*, antifungal activity, wheat seed, sustainable plant protection

## Abstract

This study analyzed the degree of fungal contamination of cereal grains and assessed the potential of gemini surfactants as antifungal agents used in seed dressing. Identification analysis based on the ITS region showed that representatives of the genera *Penicillium* and *Fusarium* were most frequently identified among the isolated microorganisms. The sensitivity testing of the gemini surfactants—12-6-12 hexamethylene-1,6-bis(N-dodecyl-N,N-dimethylammonium bromide) and 12-O-12 3-oxa-1,5-pentane-bis(N-dodecyl-N,N-dimethylammonium bromide), as well as formulations derived from them—showed that the growth of mold monocultures was inhibited at concentrations <0.0005–0.0016%. A consortium containing a mixture of five different strains inhibited the growth at concentrations of 0.031–0.125%. In studies of treated grains, both on synthetic media and in pot tests, the following parameters were considered: the type of gemini surfactant in the fungicide, the coating agent concentration, and various filamentous fungi. It was found that wheat grains showed comparatively lower infection levels under the tested conditions for the 12-6-12/N/IT7/S formulation, containing the main active ingredient, hexamethylene-1,6-bis(N,N-dimethyl-N-dodecylammonium) dibromide, and a single concentration of the coating agent. *Fusarium* sp. monocultures colonized treated grain more quickly than a consortium of five strains. The results indicate that seed coatings based on gemini surfactants may represent a potentially useful approach under controlled conditions; however, further studies including toxicity assessment, environmental impact evaluation, and field validation are required.

## 1. Introduction

Agriculture has been the foundation of human civilization for centuries, providing food and raw materials for developing societies. As the global population grows, so do the demands for increased agricultural productivity and food security. One of the main challenges of modern agriculture is effectively protecting crops from various biological threats, primarily filamentous fungi, which can drastically reduce the quality and quantity of yields [1,2].

Seed grain, the basis of plant production, is particularly susceptible to fungal infections, both during storage and in the initial stages of germination during cultivation. The presence of molds can lead to reduced seed quality and, consequently, reduced germination capacity and significant yield losses [1]. Therefore, effective seed grain protection is a key element in ensuring stable and efficient agricultural production.

Wheat (*Triticum aestivum* L.) is of unquestionable importance in global crop production. It is one of the most important cereal crops grown worldwide and constitutes a staple diet in many countries. The high economic value of this plant makes maintaining the appropriate seed quality crucial for the sustainability of agricultural production. However, wheat seeds are susceptible to infections caused by various fungal pathogens, which can develop both during plant growth in the field and during seed storage. The most common pathogens include species of the genera *Fusarium*, *Alternaria*, *Aspergillus*, and *Penicillium*, which lead to reduced germination, reduced grain quality, and the production of mycotoxins that pose a threat to human and animal health.

Chemical pesticides are widely used to control disease development in crops. The introduction of synthetic pesticides in the 20th century significantly influenced the development of modern agriculture, enabling the effective control of many plant pathogens [3]. Their high effectiveness in combating cereal pathogens makes these preparations essential tools in modern plant protection systems [4]. Fungicides constitute an important group, playing a key role in protecting plants against diseases caused by fungi. However, the chemical protection methods used so far, despite their high effectiveness, do not always meet the growing demands for sustainable development, and their intensive use can also lead to the emergence of resistant pathogens [5].

Faced with the challenges associated with the use of traditional fungicides, scientists are increasingly seeking alternative plant protection methods that are both effective and more environmentally friendly. One promising research direction is based on gemini surfactants, synthetic chemical compounds characterized by a specific molecular structure. They consist of two hydrophilic heads and two hydrophobic chains connected by a spacer, which gives them unique physicochemical properties. Compared to traditional monomeric surfactants, they exhibit significantly higher surface activity and lower critical micellization concentrations. Focusing on their biocidal activity at relatively low concentrations has been suggested as a potential approach to reduce the environmental burden associated with the use of plant protection products [6,7]. Due to their structure and surface-active properties, these compounds have been proposed as potential components of modern plant protection strategies. They can form stable layers on the surface of seeds, limiting the development of fungal pathogens and potentially reducing the need for high doses of conventional chemicals. Additionally, the possibility of modifying their chemical structure allows the design of compounds with increased biodegradability, which has been suggested to potentially limit their environmental impact compared to traditional pesticides [8,9].

The scientific literature has reported on the potential applications of gemini surfactants in various industrial sectors. These compounds exhibit biocidal properties, including antibacterial and antifungal activity, making them attractive tools in the control of microorganisms. Studies on the physicochemical properties of gemini surfactants have indicated their ability to form stable emulsions and suspensions, which promotes good adhesion to surfaces, including biological materials, and may contribute to their antimicrobial effectiveness [10,11,12].

The aim of this study was to assess the potential of gemini surfactants contained in prepared formulations to evaluate their potential for application in wheat seed treatment against fungal infections. Specifically, a comparison of the effectiveness of selected types of gemini surfactants in inhibiting fungal growth was conducted, ranging from tests in synthetic media to tests in soil samples. The obtained results may contribute to expanding knowledge on the use of these compounds as more environmentally friendly alternatives compared to conventional fungicides and support the development of potentially improved plant protection strategies.

## 2. Results

### 2.1. Quantitative Analysis

**Figure 1:**
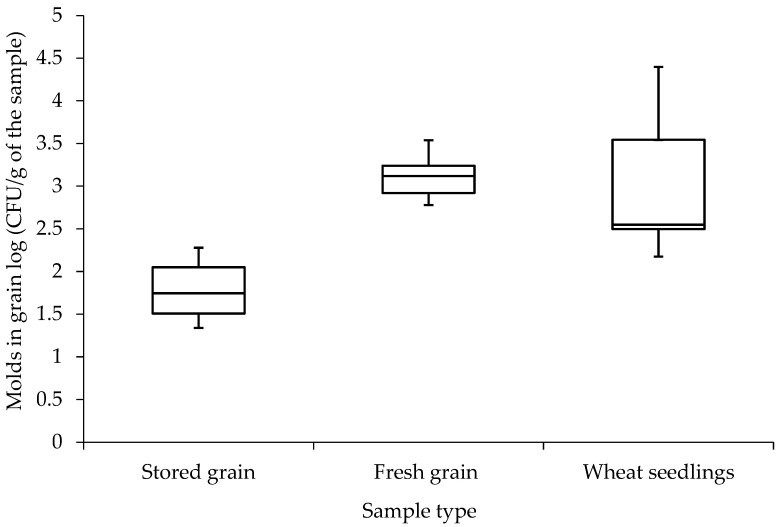
Levels of mold presence depending on the source material.

In the first stage of the research, a quantitative analysis of the content of filamentous fungi was performed both in grains (fresh, immediately after harvest and stored for 6 months in 50–70% humidity conditions) and in young seedlings taken directly from the fields. The mold amount varied depending on the raw material. However, it could be observed that samples of fresh grain and seedlings were characterized by a higher amount of mold by up to two logarithmic orders than stored grains—3.1 and 2.7 log (CFU/g) for fresh samples compared to 1.7 log (CFU/g) for stored samples (Figure 1).

### 2.2. Identification of Isolates

From the samples of both grain and seedlings obtained for quantitative analysis, individual strains of molds were isolated. Based on macroscopic observations of colonies and light microscopy images of the isolates obtained, the most frequently identified genera were selected and then subjected to genetic identification (Table 1). The analysis indicated that among the selected isolates, molds from the genera *Aspergillus* and *Penicillium* were most frequently identified in stored grain while isolates from fresh grain more often included fungi from the genera *Fusarium* and *Trichoderma*. The cultivable isolates from wheat seedlings were more diverse. In addition to the genera mentioned above, *Apiospora*, *Epicoccum*, and *Sarocladium* were also identified in these samples.

**Figure 2:**
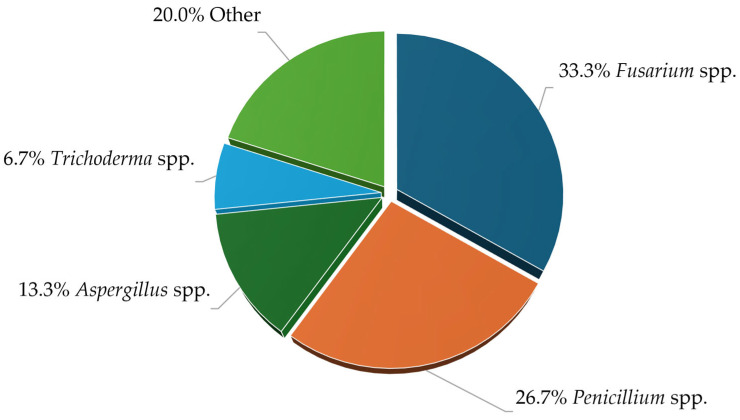
Share of genera in the isolates pool.

Considering the qualitative composition of all analyzed samples (Figure 2), the most frequently identified genera among the isolates, cultured under given condition, were *Penicillium* and *Fusarium*. Storage molds, typically associated with post-harvest contamination and quality deterioration, were primarily represented by *Aspergillus* spp. and *Penicillium* spp. However, the high presence of *Fusarium* species in fresh samples is particularly concerning, as many members of this genus are recognized plant pathogens that can infect various crops, not just the wheat studied in this case.

### 2.3. Minimal Inhibitory Concentrations and Growth of Molds in the Presence of Fungicides

The mold strains isolated and identified in the initial stage of work were used in the subsequent stages as environmental microorganisms against which the effectiveness of fungicides containing gemini surfactants as active substances was tested. In this study, two cationic compounds were used: 12-6-12 hexamethylene-1,6-bis(N-dodecyl-N,N-dimethylammonium bromide) and 3-oxa-1,5-pentane-bis(N-dodecyl-N,N-dimethylammonium bromide) 12-O-12. The preparation also included IT7 (poly(oxy-1,2-ethanediyl), alpha-tridecyl-omega-hydroxy-, branched), S (organically modified polysiloxane), and N (N-(3-aminopropyl)-N-dodecylpropane-1,3-diamine) compounds in various proportions. For the prepared fungicides, their minimum concentrations (MICs) inhibiting mold growth were determined. The determined values showed that the strains of the *Aspergillus* genus (*A. flavus*, *A. fumigatus*), as well as *F. verticillioides*, showed the lowest sensitivity to all tested substances, and their MICs ranged from 0.008% to 0.016% (Table 2). In the case of *F. graminearum* (all samples) and *A. niger* (N and N/S), substances from group 12-O-12 had lower MICs values compared to substances from group 12-6-12. The opposite situation was observed for *P. commune* (without P and N/S), *S. strictum*, and *A. fumigatus* (without IT7).

Comparing the MICs obtained for environmental strains with those obtained for the strain from the culture collection, it can also be noticed that the inhibitory concentration values were higher for the first group.

In the next stage of the study, the effect of varying concentrations of excipients was evaluated. In this case, only a mixed microbiological culture was used (Table 3). This was due to the results obtained above (Table 2) and the fact that mixed cultures of microorganisms are most common in the natural environment. For variants containing two and three times higher concentrations of non-ionic surfactants (2 and 3 IT7/S) and silicones (2 and 3IT7/S), for both the 12-O-12 and 12-6-12 groups, the MIC values were identical (0.016%) and 2–8 fold lower than the MIC values obtained for fungicides containing lower additive concentrations (IT7 and IT7/S) (Table 3).

For variants containing N-(3-aminopropyl)-N-dodecylpropane-1,3-diamine (N), only 3N/S samples containing 12-6-12 or 12-O-12 showed an MIC value of 0.016%. Two- and three-fold increases in the concentration of silicone S, which were intended to facilitate grain coating, showed no change in the MIC value.

Based on the obtained results, it was concluded that the addition of two compounds, IT7 and N, which could enhance antifungal activity, did not significantly reduce the concentrations inhibiting mold growth. Therefore, mixtures containing gemini surfactants 12-6-12 or 12-O-12 at a concentration of 0.125% and IT7, N, and S each at concentrations of 1% were used for further studies. In four-day cultures of the mold consortium and monocultures of *F. graminearum* and *F. verticiloides* in the presence of fungicides, their growth potential was monitored.

**Figure 3:**
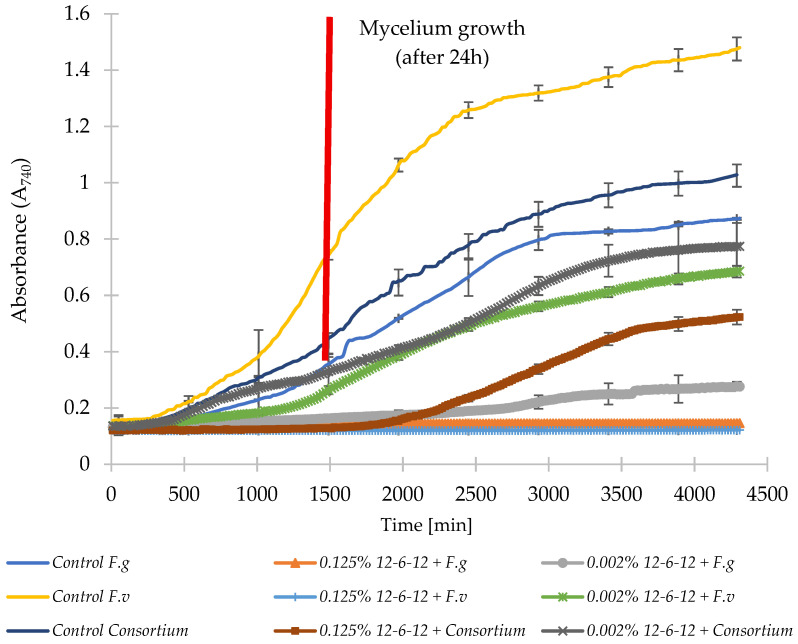
Growth curves of tested molds and consortium under different concentrations of 12-6-12/N/IT7/S. *F.g*—*Fusarium graminearum*, *F.v*—*Fusarium verticillioides*, Consortium—*F. verticillioides*, *F. graminearum*, *A. niger*, *A. flavus*, *P. commune*.

**Figure 4:**
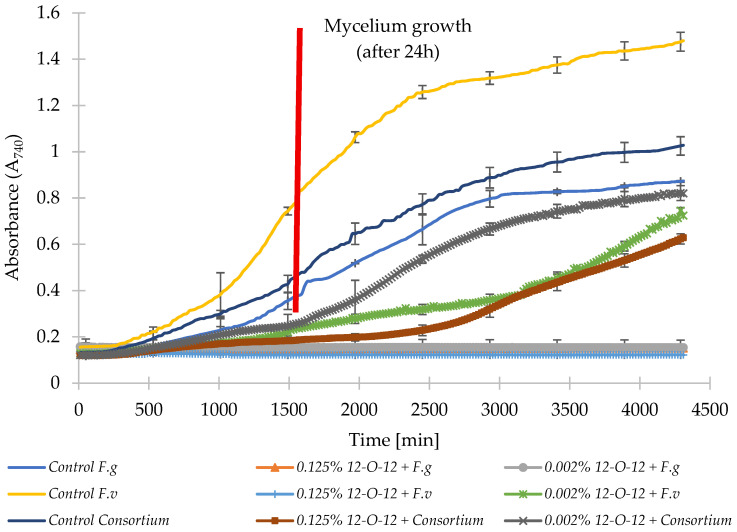
Growth curves of tested molds and consortium under different concentrations of 12-O-12/N/IT7/S. *F.g*—*Fusarium graminearum*, *F.v*—*Fusarium verticillioides*, *Consortium*—*F. verticillioides*, *F. graminearum*, *A. niger*, *A. flavus*, *P. commune*.

Across all tested variants, control samples containing untreated strains showed the fastest increases in absorbance (Figure 3 and Figure 4). Both surfactants inhibited fungal growth in a clear concentration-dependent manner. In *F. graminearum*, fungicides with gemini surfactants 12-6-12 or 12-O-12, even at the lowest concentrations of 0.002%, reduced the growth of biomass. *F. verticillioides* showed higher tolerance, but both surfactants limited its growth. In the control sample, after 24 h (1440 min), absorbance reached 0.72, with the addition of 12-6-12 A = 0.25, and in the sample with the addition of 12-O-12 A = 0.21, which clearly indicates a delay in the germination process leading to mycelium development. The growth of all monocultures treated with fungicide at the higher concentration of 0.125% was completely inhibited.

In the mixed fungal consortium, both surfactants limited biomass growth proportionally to concentration, but greater variability was observed here than in the monocultures. Both fungicides at 0.002% concentrations limited mycelial growth in the mixed culture of five mold strains. After 24 h (1440 min), the control sample had an absorbance of 0.41. In samples containing gemini surfactants at 0.002% concentrations, the absorbance was 0.26 for 12-6-12 and 0.24 for 12-O-12. This increase was twice that observed at the beginning of the experiment (time zero), indicating microbial growth. In contrast, the sample containing the fungicide 12-6-12 at a concentration of 0.125% showed no increase in absorbance, while the sample containing 12-O-12 showed an increase of 0.05%. Based on the data in Figure 3 and Figure 4, a slight increase in biomass was observed after 2500 min (i.e., almost 42 h), indicating the good mold-inhibiting properties of both fungicides.

### 2.4. Seed Treatment of Wheat Grain with Fungicides Based on Gemini Surfactants—Laboratory Trials

Wheat grain studies were conducted using two varieties: Artist and Euforia. Grains were treated with fungicides containing the gemini surfactants 12-6-12 or 12-O-12 at 0.125% concentrations and soaked in the solution for 1 h. The basic amount of grain coating agent S and a twice-as-large dose of 2S were used. Samples prepared in this way were exposed to a monoculture of *F. graminearum* and a consortium containing five different mold strains: *F. verticillioides*, *F. graminearum*, *A. niger*, *A. flavus*, and *P. commune.* After 2, 3, 4, 7, and 14 days, the percentages of grains infected by molds were determined. Factorial ANOVA revealed highly significant effects of all tested factors (variety, pathogen, surface disinfection, and treatment) and their interactions (all *p* < 0.001), indicating a strongly context-dependent response. Among these factors, the pathogen factor exerted the strongest effect (F(2, 120) = 579.87, *p* < 0.001), reflecting major differences between monoculture, consortium, and pathogen-free conditions.

**Figure 5:**
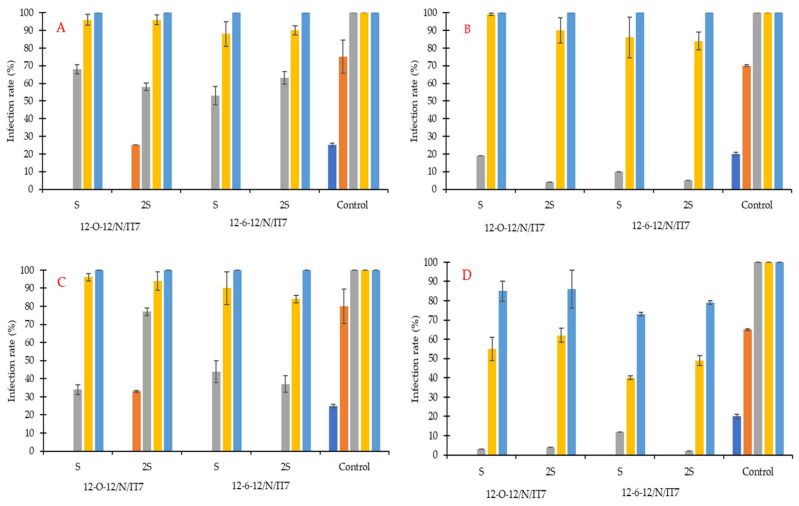
Infection rate (in %) of wheat variety Euforia by (**A**,**C**)—monoculture of *F. graminearum* and (**B**,**D**)—consortium of molds in 2 types of grain: (**A**,**B**)—sterile wheat grain and (**C**,**D**)—non-sterile wheat grain, all treated for 1 h by fungicides. Times of incubation: ● 2 days, ● 3 days, ● 4 days, ● 7 days, and ● 14 days. Tested compounds: 12-6-12 hexamethylene-1,6-bis(N-dodecyl-N,N-dimethylammonium bromide); 12-O-12 3-oxa-1,5-pentane-bis(N-dodecyl-N,N-dimethylammonium bromide); IT7—poly(oxy-1,2-ethanediyl), alpha.-tridecyl-omega.-hydroxy-, branched; S—coating agent, organically modified polysiloxane; N—N-(3-aminopropyl)-N-dodecylpropane-1,3-diamine.

The first grains of wheat cultivar Euforia not treated with preparations based on gemini surfactants (control sample) were infected at the level of 25% by *F. graminearum* and 20% by a mold consortium containing *A. niger*, *F. verticillioides*, *F. graminearum*, *P. commune*, and *A. flavus* after only 2 days, regardless of whether disinfected (treated with sodium hypochlorite NaOCl—Figure 5A,B) or non-disinfected (Figure 5C,D) grains were tested. After another day, this level increased to 75 and 80% for samples with monoculture on disinfected and non-disinfected grains, respectively. For the mold consortium, these results were 70% for surface-disinfected grain (Figure 5B) and 65% for non-disinfected grain (Figure 5D). In agreement with the statistical analysis (Supplementary Material S1), the pathogen type significantly influenced infection dynamics, with monoculture conditions consistently resulting in higher infection levels than the consortium (*p* < 0.001). Differences related to disinfection were also significant (F(1, 120) = 203.40, *p* < 0.001), although their direction varied depending on treatment and pathogen. In the initial incubation phase, the *F. graminearum* monoculture consistently colonized grains faster and to a greater extent than the five-strain consortium, as indicated by the statistical comparisons. These differences became less apparent on the fourth day of the experiment, when complete (100%) infection of the test material was observed in all control samples. Descriptive statistics confirmed that untreated controls consistently exhibited the highest infection levels (typically ~85–100%).

In samples treated with biocides, the first symptoms of *F. graminearum* infection were visible after 3 days only for biocides containing the active ingredient 12-O-12, but only in samples where a higher amount of organically modified polysiloxane (2S) was used. In this case, 25% of surface-disinfected grains (after pre-treatment with NaOCl) and 33% of untreated grains were infected. Post hoc comparisons confirmed that all treatments significantly reduced infection relative to the control (*p* < 0.001), with 12-6-12/S showing the strongest effect, followed by 12-6-12/2S, while both 12-O-12 variants were less effective (*p* ≤ 10^−6^ for surfactant type differences). Based on the results, it can be assumed that a higher amount of coating substance did not result in a consistent improvement in antifungal efficacy. Although differences between S and 2S were observed in specific cases, these effects were dependent on interactions with other factors, particularly the pathogen and disinfection, as confirmed by significant higher-order interactions (all *p* < 0.001).

Considering all variables—including biocide type, coating agent concentration (S vs. 2S), surface disinfection with sodium hypochlorite, and mold composition—the Euforia grain tended to show lower infection levels in treatments involving the 12-6-12 formulation, particularly in the S variant. Across all treatments, grains were more readily colonized under monoculture conditions, followed by the consortium, with the lowest infection levels observed under pathogen-free conditions (*p* < 0.001).

**Figure 6:**
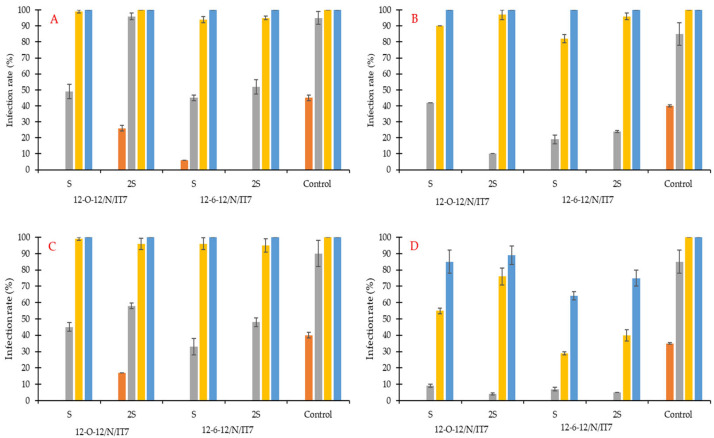
Infection rate (in %) of wheat variety Artist by (**A**,**C**)—monoculture of *F. graminearum* and (**B**,**D**)—consortium of molds in 2 types of grain: (**A**,**B**)—sterile wheat grain and (**C**,**D**)—non-sterile wheat grain, all treated for 1 h by fungicides. Times of incubation: ● 2 days, ● 3 days, ● 4 days, ● 7 days, and ● 14 days. Tested compounds: 12-6-12 hexamethylene-1,6-bis(N-dodecyl-N,N-dimethylammonium bromide); 12-O-12 3-oxa-1,5-pentane-bis(N-dodecyl-N,N-dimethylammonium bromide); IT7—poly(oxy-1,2-ethanediyl), alpha.-tridecyl-omega.-hydroxy-, branched; S—coating agent, organically modified polysiloxane; N—N-(3-aminopropyl)-N-dodecylpropane-1,3-diamine.

In the case of the Artist wheat cultivar (Figure 6), the first grains in the control samples (untreated) were infected only after 3 days, which was 24 h later than in the Euforia cultivar. The percentages of grains infected by the *F. graminearum* monoculture were 40% and 45% for non-disinfected and disinfected samples, respectively, and 40% and 35%, respectively in the case of grains infected with a mixture of five mold strains. At the same time, the first infections were also recorded in grains treated with 12-O-12/2S, in both disinfected (26%) and untreated (17%) variants. After 7 days, all untreated grains were 100% infected by the fungus. Similarly to Euforia, statistical analysis confirmed significant effects of variety (F(1, 120) = 67.25, *p* < 0.001) and its interactions with other factors (e.g., variety × pathogen: F(2, 120) = 71.30, *p* < 0.001); the response to treatments differed between cultivars depending on pathogen and environmental conditions.

For the Artist variety, differences between S and 2S were detected in selected cases; however, these effects were not consistent across experimental conditions. Treatment effects depended strongly on the pathogen type and disinfection conditions rather than the coating agent concentration alone. At day 4, differences between S and 2S variants were observed in selected treatment combinations. At later incubation times, infection ranges between S and 2S variants largely overlapped. Across different wheat varieties, grain disinfection conditions, and mold systems, infection levels were similar between the double-coating agent concentration (2S) and the single concentration (S).

In most cases, for the monoculture after 7 days of incubation and for the mold consortium after 14 days of incubation (Figure 5 and Figure 6), grain treated with 12-6-12 group variants showed infection levels ranging from 63 to 79%, whereas grain treated with 12-O-12 group variants showed infection levels in the range of 85–89% at the corresponding time points. The lowest infection level recorded after 14 days of incubation was 63%, observed for the 12-6-12-S variant where non-disinfected grain of the Artist variety was tested against the mold consortium.

### 2.5. Seed Treatment of Wheat Grain with Gemini Surfactants—Soil Trials

Based on the previous results, in the final stage of the study, when the seeds were placed in the soil, two fungicides were used for dressing—containing one of the gemini surfactants 12-6-12 or 12-O-12, as well as IT7, S, and N in each formulation. Seeds were sown in soil containing molds: monocultures of *F. graminearum* or *F. verticiloides* and a consortium of five mold strains. Sterile soil was also used in the study. Seed germination was assessed after 3 days (shown in %, Table 4 and Table 5), and growth analysis was performed after 9 days of incubation. These parameters were also used as indicators of potential inhibitory effects of the tested formulations in relation to plant growth. Statistical analysis revealed significant effects of variety, treatment, and their interactions across all plant compartments, with patterns differing between above-ground tissues, roots, and whole-plant responses (Supplementary Material S2).

Germination results assessed after 3 days showed clear differences between varieties, pathogen conditions, and treatments (Table 4 and Table 5). In the Euforia variety, untreated seeds in sterile soil exhibited high germination (85.0 ± 4.0%), which further increased slightly after treatment with both 12-6-12 (90.0 ± 2.0%) and 12-O-12 (93.0 ± 3.0%). In contrast, the presence of pathogens reduced germination in untreated samples, particularly in the consortium (44.0 ± 6.0%), followed by *F. verticillioides* (66.0 ± 6.0%) and *F. graminearum* (71.0 ± 3.0%). The application of both surfactant-based treatments markedly improved germination under all pathogen conditions, reaching 97.0% for *F. graminearum* and approximately 82–89% for *F. verticillioides* and consortium treatments. In the Artist variety, germination remained consistently high across all conditions, ranging from 92.0 to 99.0% in sterile soil and from 95.0 to 99.0% in pathogen-treated samples. The presence of pathogens did not reduce germination to the same extent as observed for Euforia. Treatment with 12-6-12 and 12-O-12 resulted in slight increases or the maintenance of high germination levels (96.0–99.0%), with the highest values observed for 12-6-12 under *F. graminearum* conditions (99.0 ± 1.0%). Pathogen presence had a stronger negative impact on germination in Euforia than in Artist while both surfactant-based treatments mitigated this effect and maintained high germination levels across most conditions.

**Figure 7:**
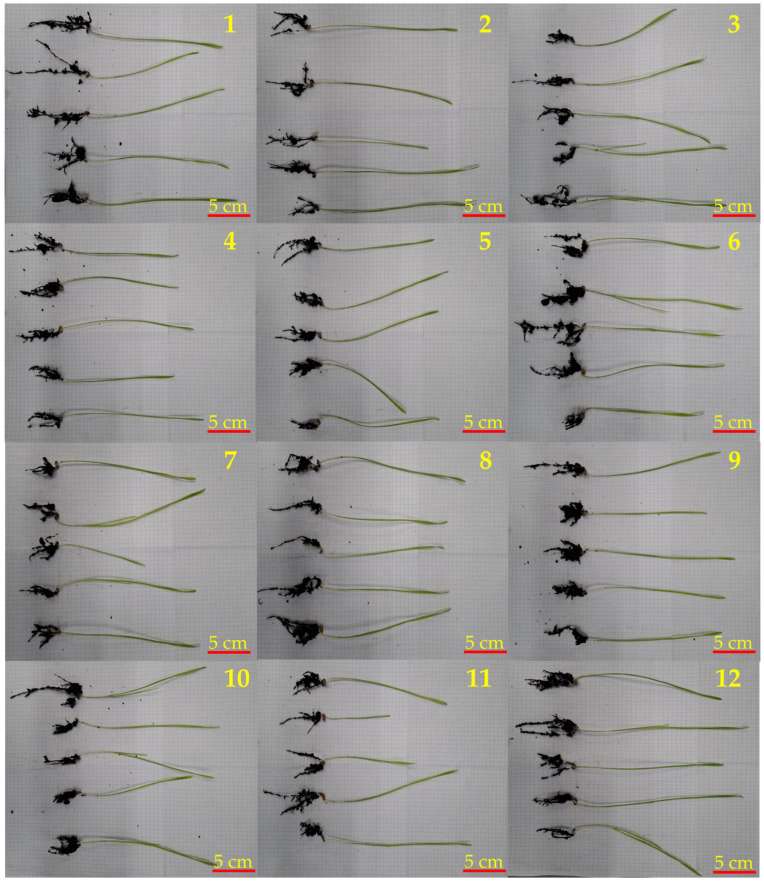
Artist grain seedlings. From the left: 1—untreated, 2—treated 12-6-12/N/IT7/S, 3—treated 12-O-12/N/IT7/S, 4—untreated + *F. graminearum*, 5—untreated + *F. verticillioides*, 6—untreated + Consortium, 7—12-O-12/N/IT7/S + *F. graminearum*, 8—12-6-12/N/IT7/S + *F. graminearum*, 9—12-O-12/N/IT7/S + *F. verticillioides*, 10—12-6-12/N/IT7/S + *F. verticillioides*, 11—12-O-12/N/IT7/S + Consortium, 12—12-6-12/N/IT7/S + Consortium.

The above-ground length of wheat grain variety *Artist* samples in soil with *F. graminearum*, treated 12-6-12/N/IT7/S (sample no. 8, Table 4; Figure 7; Supplementary Material S2), showed significantly better growth—mean: 19.3 cm, compared to the untreated grain (sample 4, mean: 15.8 cm). A similar relationship was observed for this fungicide in the case of seeds placed in soil with the addition of *F. verticillioides*—sample no. 10 in comparison to no. 5—where the above-ground length was 4.4 cm higher. These observations are consistent with ANOVA results showing significant treatment effects on above-ground growth (F(2, 96) = 17.36, *p* < 0.001), as well as significant interactions between the variety and pathogen factors (F(3, 96) = 4.20, *p* = 0.008) and a strong three-way interaction (F(6, 96) = 7.46, *p* < 0.001). Post hoc comparisons confirmed that 12-6-12/N/IT7/S significantly increased above-ground performance relative to untreated controls, particularly under *F. graminearum* infection (*p* ≤ 0.005). In a consortium of five mold strains (sample 12), the above-ground length was 1.1 cm longer compared to the untreated sample (no. 6), but these differences were not statistically significant, reflecting the context-dependent nature of treatment effects. In the case of differences in root length, the fungicide with 12-6-12 in soil with *F. verticillioides* sample no. 10 (mean: 6.8 cm) showed a trend toward increased root length compared to untreated grain sample no. 5 (mean: 4.9 cm). Although such differences were not always significant in pairwise comparisons, ANOVA indicated significant main effects of variety (F(1, 96) = 28.03, *p* < 0.001) and treatment (F(2, 96) = 7.44, *p* < 0.001), as well as significant interactions (*p* ≤ 0.023). Post hoc analysis confirmed that 12-6-12 significantly improved root performance compared with no treatment, particularly in the Artist variety and under *F. graminearum* conditions (*p* ≤ 0.005). The whole plant length, after the treatment of grains by fungicide 12-6-12/N/IT7/S in soil with *F. verticillioides* (mean: 24.6 cm, sample no. 10), was higher than in the untreated grain no. 5 sample (mean: 18.3 cm). Furthermore, fungicide with 12-6-12/N/IT7/S in soil with *F. graminearum* showed an increase in the length of the whole plant (mean: 27.5 cm), indicating differences between treatments under these conditions. These findings are consistent with robust analysis results showing highly significant effects of variety (F(1, 96) = 156.11, *p* < 0.001), treatment (F(2, 96) = 33.01, *p* < 0.001), and their interactions for whole-plant responses, with treatment effects following a clear hierarchy: 12-6-12/N/IT7/S > 12-O-12/N/IT7/S > control (all *p* ≤ 0.003).

**Figure 8:**
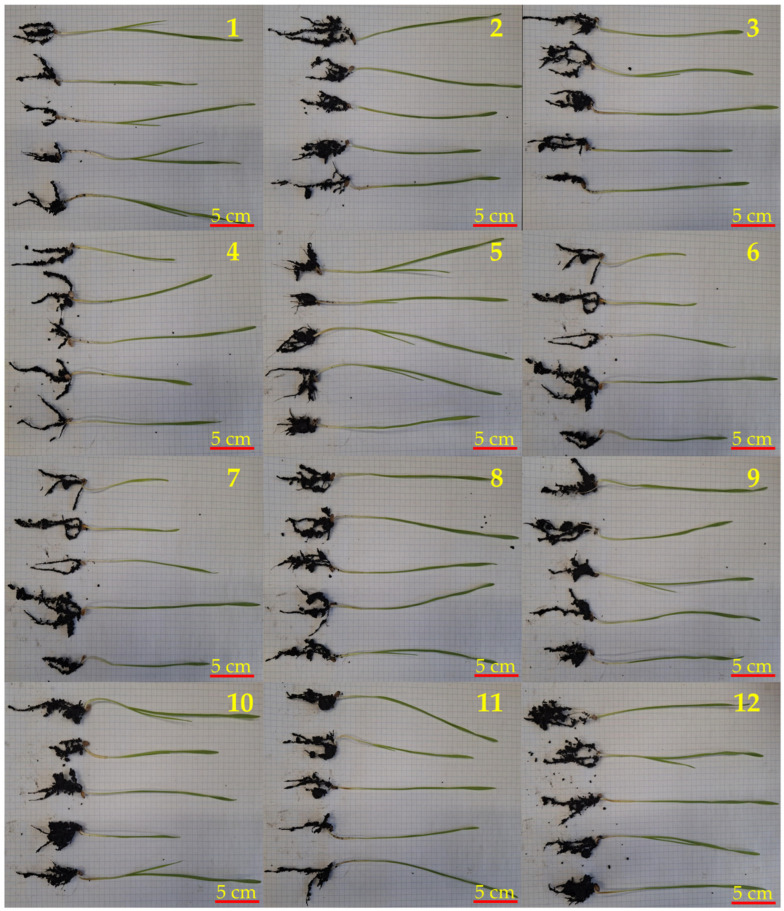
Euforia grain seedlings. From the left: 1—untreated, 2—treated 12-6-12/N/IT7/S, 3—treated 12-O-12/N/IT7/S, 4—untreated + *F. graminearum*, 5—untreated + *F. verticillioides*, 6—untreated + Consortium, 7—12-O-12/N/IT7/S + *F. graminearum*, 8—12-6-12/N/IT7/S + *F. graminearum*, 9—12-O-12/N/IT7/S + *F. verticillioides*, 10—12-6-12/N/IT7/S + *F. verticillioides*, 11—12-O-12/N/IT7/S + Consortium, 12—12-6-12/N/IT7/S + Consortium.

In Euforia grain (Table 5, Figure 8) the highest above-ground growth was observed in untreated grain (mean: 18.2 cm) and 12-6-12/N/IT7/S + Consortium (mean: 18.2 cm). Both combinations showed significantly greater shoot length values compared to the untreated grain + *F. graminearum* (mean: 11.9 cm) and untreated grain + Consortium (mean: 11.9 cm) combinations. The presence of *F. graminearum* significantly limited shoot growth; however, the application of 12-6-12/N/IT7/S (mean: 16.5 cm) was associated with higher shoot lengths under pathogen stress conditions, consistent with the significant treatment effects detected for above-ground tissues. Regarding root length, the highest value was recorded for untreated grain + 12-6-12/N/IT7/S (mean: 6.8 cm), which was significantly higher than in most other treatments, including untreated grain (mean: 3.7 cm) and untreated grain + *F. graminearum* (mean: 3.9 cm). This result supports the statistically confirmed positive effect of 12-6-12 on root development. Shorter root length values in treatments involving pathogens or the consortium, such as untreated grain + *F. verticillioides* (mean: 4.4 cm) and untreated grain + Consortium (mean: 5.4 cm), indicated a trend toward reduced root growth, although differences were not statistically significant, highlighting a potential negative impact of biological factors (pathogenic molds). With respect to total plant length, the highest values were observed in 12-6-12/N/IT7/S + Consortium (mean: 23.5 cm), and these were significantly higher than in untreated grain + *F. graminearum* (mean: 15.8 cm) and untreated grain + Consortium (mean: 17.3 cm). Similarly high total growth was observed in grain + 12-6-12 (mean: 22.4 cm) and 12-6-12/N/IT7/S + *F. graminearum* (mean: 22.1 cm). In contrast, untreated grain + *F. graminearum* (mean: 15.8 cm) exhibited the lowest total length, emphasizing the strong negative effect of this pathogen on plant growth. These results are consistent with the dominant effect of variety and treatment on whole-plant responses, as well as the strong interaction effects identified in the statistical analysis.

## 3. Discussion

The quantitative analysis in this study highlights notable variations in mold presence across different raw materials, indicating a significant influence of the material’s condition on mold proliferation. Fresh grains and seedlings demonstrated substantially higher levels of mold presence, with log (CFU/g) values of 3.1 and 2.7, respectively, compared to stored grains, which showed a log (CFU/g) of 1.7. This phenomenon likely occurred due to high residual water content in fresh samples compared to grain obtained from storage conditions, where humidity was significantly lowered (12–14% in storage compared to 18–25% at harvest). These results align with the findings of Wang et al. (2020) and Wen et al. (2020), who observed elevated fungal loads in freshly harvested cereal grains due to higher water activity and the presence of easily metabolizable substrates [13,14]. Similarly, Birck et al. (2006) reported that grain drying during storage leads to a gradual reduction in microbial viability, which is consistent with the decline in fungal counts observed in our research [15]. However, Molina-Herrera et al. (2025) noted that in some storage systems with suboptimal aeration, mold levels may increase again after prolonged storage, suggesting that environmental control rather than storage duration alone determines fungal stability [16].

The identification of molds by the microscopic and nucleotide sequence analysis of the ITS1/ITS2 region revealed that *Penicillium* and *Fusarium* were the predominant genera under the studied conditions. These play a significant role in pre- and post-harvest spoilage and mycotoxin production [17]. The detection of *Aspergillus*, *Apiospora*, *Epicoccum*, and *Sarocladium* further illustrates the diversity of the fungal community within the grain samples, reflecting a broad spectrum of mold species capable of thriving in such environments. The presence of *Fusarium* and *Aspergillus*, particularly, is concerning due to its association with mycotoxin contamination, a well-documented issue in stored grains, as discussed by Atalla et al. (2003) and Johns et al. (2022) [18,19]. The occurrence of *Epicoccum* and *Sarocladium* alongside *Fusarium* and *Aspergillus* in plant-associated substrates and co-occurrence/network analyses often shows negative correlations consistent with competitive interactions, suggesting these genera may influence *Fusarium* abundance and mycotoxin dynamics in some systems [20,21]. It should be also noted that these results reflect only cultivable fungi under the applied conditions and do not fully represent total fungal diversity.

Minimal inhibitory concentration (MIC) assessments for antifungal substances against both reference and isolated strains revealed varying levels of sensitivity. *Aspergillus flavus*, *Aspergillus fumigatus*, and *Fusarium verticillioides* exhibited the lowest sensitivity to tested compounds, with MIC values ranging from 0.008% to 0.016%. These results are consistent with Borman et al. (2017), who reported their inherent resistance to a range of antifungal compounds [22]. Furthermore, the elevated resistance observed in these strains emphasizes the necessity for combination treatments or increased doses to achieve effective control, as highlighted by Jørgensen et al. (2018) and van den Bosch et al. (2011) [23,24]. When comparing MICs of environmental isolates with those of ATCC reference strains, a clear increase in resistance was observed among environmental samples—MIC values were up to 16 times higher. This observation suggests that isolates from natural or agricultural environments have developed greater tolerance, likely as a result of prolonged exposure to environmental stressors and low doses of biocides. Similar trends were documented by Arastehfar et al. (2020), who demonstrated that environmental fungal isolates often display enhanced resistance due to selective pressures in soil or storage conditions, leading to more robust and adaptable phenotypes [25]. In the earlier publications, the authors of this article, Koziróg and Brycki (2015), also emphasized the importance of conducting MIC tests on environmental isolates, given that biocides at sublethal concentrations may promote adaptive resistance and cross-resistance to other stressors [26].

In the next stage, the effect of using fungicides with gemini surfactants on the growth and development of monocultures of the *Fusarium* genus and a mixture of five mold strains was determined. Mycelium development was observed in all three control samples after 24 h. The addition of 12-O-12 and 12-6-12 compounds, even at sub-MIC concentrations of 0.002%, delayed spore germination leading to mycelium development, with stronger inhibition for *F. graminearum*. However, the results from grain experiments indicate that these effects were strongly context-dependent, as confirmed by factorial ANOVA showing significant effects of pathogen system and interactions with treatment. Comparable observations—where surfactants inhibit, but do not always completely eliminate, *Fusarium* spp. growth—had been reported for in vitro biosurfactant treatments [27,28]. Using sublethal concentrations may increase the risk of resistance development. This gentle but continuous action of the selection agent slows the growth of sensitive cells, giving an advantage to resistant mutants [29,30]. The higher infection levels observed under monoculture compared to consortium conditions are consistent with statistical results indicating that the pathogen type was the dominant factor shaping responses. Mixed communities exhibited greater variability and lower sensitivity, aligning with previous findings that microbial consortia can display broader tolerance ranges [31,32].

For the wheat grain varieties Euforia and Artist, grains without NaOCl disinfection were generally less infected than their disinfected counterparts. This observation supports the hypothesis that preserving the natural microbial community on grains may offer protective benefits, possibly through competitive exclusion or the presence of beneficial microbes that inhibit mold growth. This idea is consistent with Chen et al. (2018), who suggested that maintaining a balanced microbial community on grain surfaces can act as a natural defense mechanism against pathogenic fungi [33]. The presence of non-pathogenic microbes on grain surfaces could reduce the incidence of fungal infections by occupying ecological niches and competing for resources. Additionally, the disinfection of grain surfaces may compromise the structural integrity, rendering grains more susceptible to mold reinfection. This phenomenon has been documented in the literature, where disinfection, despite being effective at eliminating existing microbial populations, can negatively affect grain depending on the dose of disinfectant and affect processes such as root and shoot development [34,35]. In the present study, the NaOCl disinfection protocol performed well in terms of microbial elimination but caused the seed germination rate to drop below 70%. Therefore, non-disinfected seeds were used in subsequent soil samples to maintain adequate germination rates.

Building on these observations, the growth assays with *Fusarium* species indicated stronger protective and stimulatory effects of 12-6-12/N/IT7/S compared to 12-O-12/N/IT7/S. Statistical analysis demonstrated significant effects of treatment, variety, and their interactions across all plant compartments. In particular, 12-6-12/N/IT7/S significantly improved above-ground and root development relative to untreated controls, especially under *F. graminearum* infection. These findings are consistent with previous reports on gemini surfactants’ dual antimicrobial and plant-supporting activity [36,37]. The enhanced performance of 12-6-12/N/IT7/S likely arises from its structural configuration, where the hexamethylene spacer confers stronger biological activity and more effective microbial membrane disruption [38,39]. These observations are consistent with other studies showing that gemini QACs exhibit superior antifungal properties compared to mono-quaternary analogs [40].

The observed improvement in growth might also be linked to enhanced seed–soil contact, water uptake, and nutrient availability, as reported for similar surfactant-based solutions [1,41]. Importantly, neither of the tested treatments negatively affected seed germination or early plant growth under these conditions as treated samples retained similar germination ability when compared to untreated controls—although this assessment was preliminary and should be verified further.

From a practical perspective, 12-6-12/N/IT7/S formulation showed greater potential than 12-O-12/N/IT7/S as a seed treatment. Surfactant-based coatings are increasingly considered as an alternative to traditional fungicides due to their ability to both limit fungal infection and enhance plant growth [2,42]; however, the environmental impact still needs to be assessed, particularly in terms of the biodegradability and ecotoxicity of such solutions [43].

However, no direct assessment of environmental impact was performed, and further studies are required to evaluate biodegradability, residue levels, and ecotoxicity.

## 4. Materials and Methods

### 4.1. Quantitative Analysis and Isolation Source

Wheat grain samples were obtained from multiple sources to ensure representative sampling across different storage and production conditions. Stored wheat samples (*n* = 6) were acquired from two commercial sources and one research facility: two samples from commercial distributor, two samples from seed selling company Anas (Anas, Poddębice, Poland), and two samples from Central Research Center for Cultivar Research (COBORU; Słupia Wielka; Wielkopolskie voivodeship). All stored samples were maintained under controlled conditions with moisture content below 13% and temperature below 20 °C prior to analysis.

Fresh wheat grain samples (*n* = 2) were collected directly from private farmers located in the Łódź voivodeship to represent recently harvested material under field conditions.

Seedling samples (*n* = 5) were collected from five independent agricultural fields operated by private farmers in the Łódź voivodeship, with one sample obtained per field location. This sampling strategy ensured representation of diverse field environments and agricultural practices within the region.

Both seedling and wheat grain samples were designated for quantitative analysis and isolation of wild strains. No chemical or thermal treatments were applied to any samples prior to analysis. This approach preserved the natural microbial communities and grain characteristics for subsequent analyses. All samples were processed immediately upon collection or retrieved from controlled storage to minimize any potential changes in sample composition or microbial populations during the experimental period. The sampling approach was consistent with commonly used practices for the assessment of fungal contamination in cereal grains [44,45].

### 4.2. Antifungal Substances

In this study, fungicidal formulations that contained a single, main active substance (AS) from the gemini surfactant group, AAAs (additional antifungal agents), and a CA (coating agent) were used. The characteristics of individual substances are presented in Table 6.

The composition of the individual mixtures is presented in Table 7. Additional antifungal agents, IT7 and N, and a coating agent, S, were used in basic concentrations (IT7; N; S) as well as in two- (2IT7; 2N; 2S) and three-fold higher (3IT7; 3N; 3S) ones.

### 4.3. Quantitative Analysis

Ten grams of the tested wheat samples were placed in 90 mL of sterile 0.85% saline solution and shaken for 10 min. Subsequently, a series of 10-fold dilutions were made, and samples were pour-plated with molten Malt Extract Agar (Merck Millipore KGaA, Darmstadt, Germany) with chloramphenicol (0.1 g/L *w*/*v*) to inhibit bacterial growth. The samples were incubated for 48 h at 28 °C and colony-forming units were counted. Results are presented in CFU/g of sample used. Colonies present on counted plates from the highest dilution were the source for the isolation of pure cultures later used in the identification step [46].

### 4.4. Identification of Isolates

Pure cultures of the isolated strains underwent genomic DNA extraction following the procedure stated in the DNA extraction kit—GeneMATRIX Plant & Fungi DNA Purification Kit (EURX^®^, Gdansk, Poland).

Identification of molds was performed based on the ITS1/ITS2 region [47,48]. The ITS regions were amplified by PCR using MJ Mini Gradient Thermal Cycler (Bio-Rad, Hercules, CA, USA) in the following conditions: an initial denaturation at 94 °C for 120 s, and 25 cycles of denaturation at 94 °C for 60 s, annealing at 50 °C for 60 s, extension at 72 °C for 60 s, and a final extension at 72 °C for 120 s. Each sample total volume was equal to 25 µL, where Polymerase Color OptiTaq Master Mix 12 µL (EURX^®^, Gdansk, Poland), nuclease free water 10.6 µL, each universal primer ITS1 and ITS4 0.2 µL, 2 µL of isolated and purified DNA. PCR products were separated in agarose gel (1% *w*/*v*) electrophoresis in 0.5×TBE buffer (Sigma-Aldrich, Merck, KGaA, Darmstadt, Germany) against Perfect PlusTM Molecular Weight Quantitative DNA Ladder (EURX^®^, Gdansk, Poland). Afterwards, samples were sequenced in Genomed (Warsaw, Poland).

The nucleotide sequences of ITS1/2 region were compared with the sequences published in the National Center for Biotechnology Information (NCBI) database, employing the BLAST+ 2.15.0 program. Nucleotide sequences were deposited in the NCBI GenBank database.

### 4.5. Minimal Inhibitory Concentrations and Growth of Molds in the Presence of Fungicides

The MIC values were determined using the 2-fold dilution method [49]. The gemini surfactant mixtures were tested against environmental strains: *Aspergillus niger*, *Fusarium verticillioides*, *Fusarium graminearum*, *Penicillium commune*, *Aspergillus flavus*, *Sarocladium strictum*, *Aspergillus fumigatus*, a consortium containing the first five mentioned strains (*A. niger*, *F. verticillioides*, *F. graminearum*, *P. commune*, *A. flavus*), and a collection reference strain, *A. brasiliensis* ATCC 16404. Spore suspensions were prepared by washing 48 h mold cultures with Tween 80 solution and standarized in the Thoma chamber. Their concentration in inoculum was 2.0 × 10^7^ spores/mL. In the next step, 1 mL of spore suspension was mixed with 1 mL of media: Malt Extract Broth (MERCK, Darmstadt, Germany) containing serial dilutions of the tested mixtures. The initial concentration of the tested mixtures of gemini surfactants equalled 1% (*v*/*v*). The final concentrations of the solutions after inoculation ranged from 0.25% to 0.0005%. Inoculated samples were incubated for 48 h at 28 °C. The results were assessed by comparison with a control sample containing a mold culture without the addition of gemini surfactants. The MICs were defined as the lowest concentrations of the compounds in which there was no visible growth of molds.

Samples prepared for MIC determination were also used to analyze mold growth using the Biolog ODIN™ System (Biolog, Inc., Hayward, CA, USA). For this purpose, prepared mixtures of medium with the tested formulations (12-6-12/N/IT7/S or 12-O-12/N/IT7/S) and inoculated with spore suspensions were transferred to a 96-well plate. Volume of 200 µL of sample was added to each well. The study was performed using a mixed fungal consortium consisting of *Aspergillus niger*, *Aspergillus flavus*, *Fusarium verticillioides*, *Fusarium graminearum*, and *Penicillium commune*, as well as monocultures of *F. graminearum* and *F. verticillioides*. Final concentrations of 12-6-12/N/IT7/S or 12-O-12/N/IT7/S in the wells ranged from 0.25% to 0.002% (*v*/*v*). Control wells contained fungal inoculum without added surfactant, uninoculated medium with added fungicide (0.25% to 0.002% (*v*/*v*)), and a blank sample containing medium only. These plates were incubated at 28 °C for 3 days at wavelength of 740 nm (minimized the impact of color and better reflected the turbidity of the hyphae) in an Biolog ODIN™ System, allowing automatic reading of results every 20 min during incubation [50].

### 4.6. Seed Treatment of Wheat Grain with Fungicides Based on Gemini Surfactants—Laboratory Trials

In order to assess the effectiveness of the fungicides used, wheat grain was treated and then exposed to mold on a synthetic medium. Two varieties of winter wheat were used: Euforia and Artist. At the beginning, wheat grain was disinfected in a 5% (*v*/*v*) sodium hypochlorite solution (NaClO) for 12 min. The grains were separated from the solution using sterile sieves and then rinsed three times with sterile water to remove the residual sodium hypochlorite. Then the grain was dried at 25 °C for 24 h. Grain samples without any preliminary disinfection process were also used. Both types of grains (sterile and non-sterile) were exposed to the tested fungicides containing gemini surfactants 12-6-12 or 12-O-12 at a concentration of 0.125% (*v*/*v*) and also N, IT7 (N-(3-aminopropyl)-N-dodecylpropane-1,3-diamine; poly(oxy-1,2-ethanediyl), alpha-tridecyl-omega-hydroxy-, branched) and two different amounts of S (organically modified polysiloxane) by 1 h. After dressing, the grain was dried in Petri dishes in 25 °C for 24 h. Next, a spore suspension with a density of 2.0×10^7^ spores/mL was prepared and spread evenly on the surface of the MEA medium. In the experiment, a monoculture of *F. graminearum* were used, as well as a consortium containing *A. niger*, *F. verticillioides*, *F. graminearum*, *P. commune*, and *A. flavus*. On the prepared surface, treated wheat grains were evenly distributed. At the same time, control samples were prepared containing grain (disinfected or non-disinfected) on MEA medium with mold spores but without fungicides. On each plate, 20 grains were placed, and five plates (total *n* = 100 grains) constituted one replicate; three independent replicates were performed for each experimental variant. Three replicates were performed for each sample of 100 grains. All samples were incubated for up to 14 days at 28 °C. After 2, 3, 4, 7, and 14 days, the number of infected grains was counted. The number of infected grains is expressed as a percentage of 100 grains. The procedure was consistent with commonly used approaches for evaluating seed infection and seed treatment efficacy under controlled laboratory conditions [51,52].

### 4.7. Seed Treatment of Wheat Grain with Fungicides Based on Gemini Surfactants—Soil Trials

Non-disinfected grain was treated with the tested fungicides containing gemini surfactants 12-6-12 or 12-O-12 at a concentration of 0.125% (*v*/*v*), with the addition of N, IT7, and S. After dressing, the grain was dried in Petri dishes for 24 h. In parallel, soil was prepared (lawn soil, 50 L bags, Rolimpex S.A., Iława, Poland) and subjected to repeated autoclaving (121 °C, 30 min) at 24 h intervals (three cycles). Each pot contained 3 kg of sterilized soil. The prepared seed was placed in the soil. Variants of the pots included non-infected soil, soil infected with monocultures of either *F. graminearum* or *F. verticillioides*, and soil infected with consortium containing *A. niger*, *F. verticillioides*, *F. graminearum*, *P. commune*, and *A. flavus* in equal volumes to simulate natural conditions. The density of spores used to infect the pots was 2 × 10^5^ spores/mL, and for each pot, 7 mL of suspension at this concentration was used. Such prepared pots were then sown with *Euforia* and *Artist* non-treated grain or grain treated with one of the GS variants (12-6-12/N/IT7/S or 12-O-12/N/IT7/S). Pots were incubated in 20 ± 2 °C under a 16 h light/8 h dark photoperiod for 14 days, with germination assessed after 3 days and growth analyzed after 9 days. The described approach is consistent with commonly used experimental systems for evaluating plant–pathogen interactions under controlled soil conditions [53].

### 4.8. Statistical Analysis

All statistical analyses were performed in R (v4.5.2) [54]. Data import and preprocessing were conducted using the readr (v2.1.5), dplyr (v1.1.4), and tidyr (v1.3.1) packages [55,56,57]. For the synthetic medium experiment, a full-factorial analysis of variance (ANOVA) was applied using the stats package [54] to evaluate the effects of variety, pathogen, sterility, treatment, and their interactions. Assumptions of normality and homogeneity of variances were verified using the Shapiro–Wilk test (stats) and Levene’s test (car v3.1-3) [58], respectively. Post hoc comparisons were performed using estimated marginal means with Tukey adjustment implemented in the emmeans package (v1.10.0) [59]. For soil experiments, factorial ANOVA models were also applied using the stats package, with the same assumption checks (stats, car). Post hoc comparisons were conducted using emmeans with Tukey-adjusted pairwise contrasts. In cases where assumptions of normality and homogeneity were violated (whole-plant responses), robust analysis was applied. Specifically, aligned rank transform (ART) ANOVA was performed using the ARTool package (v0.11.1) [60], allowing inclusion of interaction terms while accommodating non-parametric data structure. *p*-values were adjusted for multiple comparisons using the Benjamini–Hochberg false discovery rate (FDR) method (stats package) [53], and statistical significance was determined at α = 0.05.

## 5. Conclusions

This study highlighted the influence of grain type and the microbial context when designing and applying antifungal treatments. The observed differences in mold susceptibility between grain types, treatment variants, and microbial consortia indicate the need for tailored approaches that take into account the specific conditions and challenges of each scenario. These findings contribute to the development of more effective strategies for mold management in agricultural systems, with potential applications in both pre-harvest and post-harvest contexts. Notably, the developed fungicide formulation (12-6-12-N/IT7/S) was associated with lower infection levels in selected treatments while containing a relatively low concentration of the active substance; this still requires further validation, including toxicity, environmental impact, and field-scale studies. Future research should continue to explore the interactions between different microbial communities and their impact on the efficacy of antifungal treatments, as well as the long-term effects of such treatments on grain quality and safety.

## Figures and Tables

**Table 1:** Identification of fungal strains isolated from wheat grains and seedlings.

No.	Strain Name	Similarity [%] *	GenBank Accession Number **	Isolation Source
1	*Trichoderma harzianum*	99.48	PZ327790	Fresh wheat grain
2	*Fusarium verticillioides*	99.40	PZ327788	
3	*Fusarium proliferatum*	99.06	PP809446	
4	*Penicillium commune*	99.26	PZ327787	Stored wheat grain
5	*Penicillium jensenii*	99.45	PP809419	
6	*Aspergillus chevalieri*	99.42	PZ327789	
7	*Fusarium avenaceum*	99.69	PP809432	Wheat seedlings
8	*Aspergillus niger*	100.00	PP809416	
9	*Penicillium griseofulvum*	99.09	PP809417	
10	*Apiospora arundinis*	99.63	PP809402	
11	*Fusarium oxysporum*	99.03	PP809444	
12	*Fusarium graminearum*	99.60	PP809435	
13	*Sarocladium strictum*	98.53	PP809409	
14	*Epicoccum nigrum*	99.41	PP809406	
15	*Penicillium raperi*	97.84	PP809421	
16	*Trichoderma hamatum*	98.60	PZ327791	

* in reference to NCBI GenBank; ** deposited in the NCBI GenBank database.

**Table 2:** Minimal inhibitory concentrations (MICs) of tested substances with gemini surfactants against used strains.

**Substance Name**	***A. niger***	***F. verticillioides***	***P. commune***	***A. flavus***	**Consortium**	***F. graminearum***	***S. strictum***	***A. fumigatus***	***A. brasiliensis*** **ATCC 16404**
	Concentration [% *v*/*v*]								
12-6-12									
P	0.004	0.008	0.004	0.016	0.031	0.008	<0.0005	0.008	0.002
IT7	0.004	0.008	0.002	0.016	0.062	0.008	<0.0005	0.008	0.002
IT7/S	0.004	0.008	0.004	0.016	0.125	0.004	<0.0005	0.008	0.002
N	0.008	0.008	0.001	0.016	0.031	0.008	<0.0005	0.008	0.002
N/S	0.016	0.008	0.004	0.016	0.016	0.004	<0.0005	0.008	0.001
12-O-12									
P	0.004	0.008	0.004	0.016	0.031	0.004	0.001	0.016	0.002
IT7	0.004	0.008	0.004	0.016	0.031	0.001	0.001	0.008	0.002
IT7/S	0.008	0.008	0.008	0.016	0.031	0.001	0.001	0.016	0.002
N	0.002	0.008	0.016	0.016	0.031	0.002	0.002	0.016	0.002
N/S	0.002	0.008	0.004	0.016	0.031	0.002	0.002	0.016	0.002

**Table 3:** Minimal inhibitory concentrations (MICs) of tested fungicides against the used mold consortium.

Variant of Additive	12-6-12	12-O-12
	Concentration [% *v*/*v*]	
2 N/S	0.031	0.031
3 N/S	0.016	0.016
N/2 S	0.031	0.031
N/3 S	0.031	0.031
2 IT7	0.016	0.016
3 IT7	0.016	0.016
2 IT7/S	0.016	0.016
3 IT7/S	0.016	0.016

Tested compounds: gemini surfactant 12-6-12 hexamethylene-1,6-bis(N-dodecyl-N,N-dimethylammonium bromide; gemini surfactant 12-O-12 3-oxa-1,5-pentane-bis(N-dodecyl-N,N-dimethylammonium bromide), where: P—pure gemini surfactant, IT7—non-ionic surfactant, poly(oxy-1,2-ethanediyl), alpha.-tridecyl-omega.-hydroxy-, branched; S—coating agent, organically modified polysiloxane; N—N-(3-aminopropyl)-N-dodecylpropane-1,3-diamine.

**Table 4:** Artist grain growth assessment after fungicide treatments under different soil conditions.

No.	Type of Grain	Above-Ground Length [cm]	Root Length [cm]	Whole Plant Length [cm]	Germination [%]
	In sterile soil				
1	Untreated	13.0 ± 2.9	7.5 ± 2.1	20.5 ± 4.0	92.0 ± 2.0
2	Treated 12-6-12/N/IT7/S	16.8 ± 1.5	6.4 ± 0.7	23.2 ± 1.7	96.0 ± 2.0
3	Treated 12-O-12/N/IT7/S	16.0 ± 1.5	5.5 ± 2.1	21.5 ± 3.4	99.0 ± 1.0
	Untreated sample in soil with molds				
4	*F. graminearum*	15.8 ± 0.9	6.1 ± 1.0	21.9 ± 1.7	95.0 ± 3.0
5	*F.* *verticillioides*	13.4 ± 1.6	4.9 ± 0.7	18.3 ± 2.2	96.0 ± 3.0
6	Consortium	16.2 ± 1.0	7.2 ± 2.0	23.4 ± 2.9	99.0 ± 1.0
	Treated samples in soil with molds				
7	12-O-12/N/IT7/S + *F. graminearum*	16.0 ± 1.1	5.6 ± 0.7	21.6 ± 1.1	98.0 ± 1.0
8	12-6-12/N/IT7/S + *F. graminearum*	19.3 ± 0.8	8.2 ± 1.4	27.5 ± 1.9	99.0 ± 1.0
9	12-O-12/N/IT7/S + *F. verticillioides*	16.3 ± 1.2	6.7 ± 1.0	23.0 ± 2.0	96.0 ± 1.0
10	12-6-12/N/IT7/S + *F. verticillioides*	17.8 ± 0.8	6.8 ± 0.8	24.6 ± 1.3	97.0 ± 2.0
11	12-O-12/N/IT7/S + Consortium	14.7 ± 3.2	4.8 ± 1.4	19.5 ± 4.6	97.0 ± 3.0
12	12-6-12/N/IT7/S + Consortium	17.3 ± 0.6	6.6 ± 1.3	23.9 ± 1.8	98.0 ± 1.0

**Table 5:** Euforia grain growth assessment after fungicide treatments under different soil conditions.

No.	Type of Grain	Above-Ground Length [cm]	Root Length [cm]	Whole Plant Length [cm]	Germination [%]
	In sterile soil				
1	Untreated	18.2 ± 2.1	3.7 ± 0.8	21.9 ± 2.3	85.0 ± 4.0
2	Treated 12-6-12/N/IT7/S	15.6 ± 1.6	6.8 ± 1.6	22.4 ± 2.0	90.0 ± 2.0
3	Treated 12-O-12/N/IT7/S	15.3 ± 1.7	5.1 ± 1.2	20.4 ± 1.5	93.0 ± 3.0
	Untreated sample in soil with molds				
4	+ *F. graminearum*	11.9 ± 2.7	3.9 ± 1.2	15.8 ± 1.5	71.0 ± 3.0
5	+ *F. verticillioides*	16.4 ± 1.4	4.4 ± 0.7	20.8 ± 1.7	66.0± 6.0
6	+ Consortium	11.9 ± 3.8	5.4 ± 1.3	17.3 ± 4.5	44.0 ± 6.0
	Treated samples in soil with molds				
7	12-O-12/N/IT7/S + *F. graminearum*	15.6 ± 2.5	5.4 ± 1.5	21.0 ± 3.0	97.0 ± 1.0
8	12-6-12/N/IT7/S + *F. graminearum*	16.5 ± 0.9	5.6 ± 0.7	22.1 ± 0.7	97.0 ± 2.0
9	12-O-12/N/IT7/S + *F. verticillioides*	13.8 ± 3.2	5.7 ± 1.6	19.5 ± 4.4	88.0 ± 5.0
10	12-6-12/N/IT7/S + *F. verticillioides*	14.9 ± 1.3	5.3 ± 1.0	20.2 ± 1.2	89.0 ± 5.0
11	12-O-12/N/IT7/S + Consortium	15.0 ± 1.8	5.0 ± 1.0	20.0 ± 2.4	82.0 ± 5.0
12	12-6-12/N/IT7/S + Consortium	18.2 ± 1.3	5.3 ± 1.0	23.5 ± 0.9	84.0 ± 6.0

**Table 6:** The main ingredients contained in fungicides.

Symbol	Compound	Concentration [%]/Purpose of Use	Manufacturer
12-6-12	Hexamethylene-1,6-bis(N,N-dimethyl-N-dodecylammonium) dibromide	9.6/AS	MDA Sp. z o.o., Swadzim, Poland
12-O-12	3-oxa-1,5-pentamethylene-bis(N,N-dimethyl-N-dodecylammonium) dibromide	9.6/AS	MDA Sp. z o.o., Swadzim, Poland
IT7	Poly(oxy-1,2-ethanediyl), alpha-tridecyl-omega-hydroxy-, branched	0.1/AAA	PCC Exol S.A., Brzeg Dolny, Poland
N	N-(3-aminopropyl)-N-dodecylpropane-1,3-diamine	0.9/AAA	ARXADA Ltd., Basel, Switzerland
S	Organically modified polysiloxane	1.0/CA	JIANGXI HITO CHEMICAL CO., Ltd., Jiujiang, China

**Table 7:** Composition of the tested fungicide samples containing gemini surfactants as main active substances.

GS in Fungicides	No.	Poly(oxy-1,2-ethanediyl), alpha.-tridecyl-. omega.-hydroxy-, Branched			Organically Modified Polysiloxane			N-(3-aminopropyl)-N-dodecylpropane-1,3-diamine		
		IT7	2IT7	3IT7	S	2S	3S	N	2N	3N
12-6-12	1									
	2	x								
	3	x			x					
	4							x		
	5				x			x		
	6				x				x	
	7				x					x
	8					x		x		
	9						x	x		
	10		x							
	11			x						
	12		x		x					
	13			x	x					
	14	x			x			x		
	15	x				x		x		
12-O-12	1									
	2	x								
	3	x			x					
	4							x		
	5				x			x		
	6				x				x	
	7				x					x
	8					x		x		
	9						x	x		
	10		x							
	11			x						
	12		x		x					
	13			x	x					
	14	x			x			x		
	15	x				x		x		

All mixtures were prepared in Department of Bioactive Products, Adam Mickiewicz University, Poznan, Poland.

## Data Availability

The original contributions presented in this study have been included in the article/Supplementary Materials. Further inquiries can be directed to the corresponding authors.

## Supplementary Materials

The following supporting information can be downloaded at: https://www.mdpi.com/article/10.3390/molecules31101568/s1, Supplementary Material S1—Synthetic medium trials: statistical data; Supplementary Material S2—Soil trials: statistical data [58,59,61].
