# Supplementary material for: Preliminary Evaluation of Gemini-Surfactant-Based Formulations for Antifungal Seed Treatment in Wheat
Source: Molecules. 2026 May 8;31(10):1568. doi: 10.3390/molecules31101568 (PMC13209164; doi:10.3390/molecules31101568)
Supplement: Supplementary file 1 [file molecules-31-01568-s001.zip › molecules-4247638-supplementary/supplementary_material_II_soil_trials.docx]

**Statistical Supplementary Material II: Soil trials**

*• Correction: Holm • α = 0.05*

# Methods

All statistical analyses were performed in R (v4.5.2; R Core Team, 2025). Experimental design was detected automatically from CSV column structure (n = 5 per group). Where a plant-part column was detected, data were stratified and each stratum analysed independently. Normality of model residuals was assessed with the Shapiro-Wilk test; variance homogeneity with Levene's test (Fox & Weisberg, 2019). Where normality was violated, five transformations were evaluated (log, square-root, arcsine, Box-Cox, reciprocal; Venables & Ripley, 2002) and the transformation producing the greatest improvement in Shapiro-Wilk p-value was selected. Where factorial residuals could not be normalised, Aligned Rank Transform ANOVA (ART-ANOVA) was applied (Wobbrock et al., 2011) with pairwise ART contrasts (Holm correction). Statistical significance was set at α = 0.05.

# Results Summary

## Soil trials [Above-ground]

Factorial ANOVA revealed significant effects of: Variety (F(1,96) =  4.9494, p = 0.0284, partial η² = 0.0490); Treatment (F(2,96) = 17.3579, p = 3.7e-07, partial η² = 0.2656); Variety:Pathogen (F(3,96) =  4.2001, p = 0.0077, partial η² = 0.1160); Variety:Pathogen:Treatment (F(6,96) =  7.4573, p = 1.4e-06, partial η² = 0.3179). Non-significant terms are omitted.

## Soil trials [Root]

Factorial ANOVA revealed significant effects of: Variety (F(1,96) = 28.0272, p = 7.6e-07, partial η² = 0.2260); Treatment (F(2,96) =  7.4410, p = 9.9e-04, partial η² = 0.1342); Variety:Treatment (F(2,96) =  4.6342, p = 0.0120, partial η² = 0.0880); Pathogen:Treatment (F(6,96) =  3.1124, p = 0.0079, partial η² = 0.1628); Variety:Pathogen:Treatment (F(6,96) =  2.5850, p = 0.0230, partial η² = 0.1391). Non-significant terms are omitted.

## Soil trials [Whole plant]

Factorial ANOVA revealed significant effects of: Variety (F(1,96) = 156.1052, p = 7.7e-22, partial η² = —); Treatment (F(2,96) =  33.0073, p = 1.2e-11, partial η² = —); Variety:Pathogen (F(3,96) =  5.2302, p = 0.0022, partial η² = —); Variety:Pathogen:Treatment (F(6,96) =  6.2827, p = 1.3e-05, partial η² = —). Non-significant terms are omitted.

# Abbreviations

| **Abbreviation** | **Definition** |
| --- | --- |
| ANOVA | Analysis of Variance |
| ART | Aligned Rank Transform (non-parametric factorial; Wobbrock et al., 2011) |
| W | Shapiro-Wilk W statistic |
| F | F-ratio |
| p | p-value |
| α | Significance level (α = 0.05) |
| df | Degrees of freedom |
| M | Mean |
| SD | Standard deviation |
| Mdn | Median |
| HSD | Tukey Honestly Significant Difference |
| BH | Benjamini-Hochberg false discovery rate |
| η² | Eta-squared: proportion of total variance explained |
| partialη² | Partial eta-squared: variance explained, partialling out other terms |
| ω² | Omega-squared: bias-corrected eta-squared |
| partialω² | Partial omega-squared: bias-corrected partial omega-squared |

# Assumptions

## Soil trials [Above-ground]

| **Test** | **Statistic** | **p** | **Conclusion** |
| --- | --- | --- | --- |
| Shapiro-Wilk (residuals) | 0.9790 | 0.0574 | Normal |
| Levene's test | 1.2162 | 0.2508 | Equal |

## Soil trials [Root]

| **Test** | **Statistic** | **p** | **Conclusion** |
| --- | --- | --- | --- |
| Shapiro-Wilk (residuals) | 0.9816 | 0.0993 | Normal |
| Levene's test | 0.5868 | 0.9278 | Equal |

## Soil trials [Whole plant]

| **Test** | **Statistic** | **p** | **Conclusion** |
| --- | --- | --- | --- |
| Shapiro-Wilk (residuals) | 0.9742 | 0.0209 | Non-normal |
| Levene's test | 1.6660 | 0.0453 | Unequal |

# Main Statistical Tests

## Soil trials [Above-ground]

| **Term** | **Df** | **df_res** | **F** | **p** | **partial_eta_sq** | **partial_omega_sq** |
| --- | --- | --- | --- | --- | --- | --- |
| **Variety** | **1** | **96** | **4.9494** | **0.0284** | **0.0490** | **0.0187** |
| **Treatment** | **2** | **96** | **17.3579** | **3.7e-07** | **0.2656** | **0.1551** |
| **Variety:Pathogen** | **3** | **96** | **4.2001** | **0.0077** | **0.1160** | **0.0455** |
| **Variety:Pathogen:Treatment** | **6** | **96** | **7.4573** | **1.4e-06** | **0.3179** | **0.1837** |
| *Bold: p < α.* | | | | | | |

### Simple Effects (significant interactions)

| **Effect** | **Contrast** | **Estimate** | **SE** | **df** | **t** | **p_adj** |
| --- | --- | --- | --- | --- | --- | --- |
| **Variety** | **Artist - Euforia \| Pathogen=F. graminearum** | **2.3667** | **0.6967** | **96** | **3.3969** | **0.0040** |

## Soil trials [Root]

| **Term** | **Df** | **df_res** | **F** | **p** | **partial_eta_sq** | **partial_omega_sq** |
| --- | --- | --- | --- | --- | --- | --- |
| **Variety** | **1** | **96** | **28.0272** | **7.6e-07** | **0.2260** | **0.1459** |
| **Treatment** | **2** | **96** | **7.4410** | **9.9e-04** | **0.1342** | **0.0695** |
| **Variety:Treatment** | **2** | **96** | **4.6342** | **0.0120** | **0.0880** | **0.0392** |
| **Pathogen:Treatment** | **6** | **96** | **3.1124** | **0.0079** | **0.1628** | **0.0684** |
| **Variety:Pathogen:Treatment** | **6** | **96** | **2.5850** | **0.0230** | **0.1391** | **0.0513** |
| *Bold: p < α.* | | | | | | |

### Simple Effects (significant interactions)

| **Effect** | **Contrast** | **Estimate** | **SE** | **df** | **t** | **p_adj** |
| --- | --- | --- | --- | --- | --- | --- |
| **Variety** | **Artist - Euforia \| Treatment=12-6-12** | **1.250** | **0.4008** | **96** | **3.1189** | **0.0048** |
| **Variety** | **Artist - Euforia \| Treatment=No treatment** | **2.075** | **0.4008** | **96** | **5.1774** | **3.7e-06** |

## Soil trials [Whole plant]

| **Term** | **Df** | **df_res** | **F** | **p** |
| --- | --- | --- | --- | --- |
| **Variety** | **1** | **96** | **156.1052** | **7.7e-22** |
| **Treatment** | **2** | **96** | **33.0073** | **1.2e-11** |
| **Variety:Pathogen** | **3** | **96** | **5.2302** | **0.0022** |
| **Variety:Pathogen:Treatment** | **6** | **96** | **6.2827** | **1.3e-05** |
| *Bold: p < α.* | | | | |

# Post-hoc Comparisons (significant at p < 0.05)

*.*

## Soil trials [Above-ground]

| **Comparison** | **Estimate** | **SE** | **df** | **t** | **p_adj** |
| --- | --- | --- | --- | --- | --- |
| **Artist Consortium 12-6-12 - Euforia Consortium No treatment** | **5.4** | **1.2068** | **96** | **4.4748** | **0.0056** |
| **Artist Consortium 12-6-12 - Euforia F. graminearum No treatment** | **5.4** | **1.2068** | **96** | **4.4748** | **0.0056** |
| **Euforia Consortium 12-6-12 - Euforia Consortium No treatment** | **6.3** | **1.2068** | **96** | **5.2206** | **2.8e-04** |
| **Euforia Consortium 12-6-12 - Euforia F. graminearum No treatment** | **6.3** | **1.2068** | **96** | **5.2206** | **2.8e-04** |
| **Euforia Consortium 12-6-12 - Artist F. verticillioides No treatment** | **4.8** | **1.2068** | **96** | **3.9776** | **0.0350** |
| **Euforia Consortium 12-6-12 - Artist No treatment No treatment** | **5.2** | **1.2068** | **96** | **4.3091** | **0.0104** |
| **Artist F. graminearum 12-6-12 - Euforia F. verticillioides 12-O-12** | **5.5** | **1.2068** | **96** | **4.5577** | **0.0041** |
| **Artist F. graminearum 12-6-12 - Euforia Consortium No treatment** | **7.4** | **1.2068** | **96** | **6.1322** | **5.3e-06** |
| **Artist F. graminearum 12-6-12 - Euforia F. graminearum No treatment** | **7.4** | **1.2068** | **96** | **6.1322** | **5.3e-06** |
| **Artist F. graminearum 12-6-12 - Artist F. verticillioides No treatment** | **5.9** | **1.2068** | **96** | **4.8892** | **0.0011** |
| **Artist F. graminearum 12-6-12 - Artist No treatment No treatment** | **6.3** | **1.2068** | **96** | **5.2206** | **2.8e-04** |
| **Artist F. verticillioides 12-6-12 - Euforia Consortium No treatment** | **5.9** | **1.2068** | **96** | **4.8892** | **0.0011** |
| **Artist F. verticillioides 12-6-12 - Euforia F. graminearum No treatment** | **5.9** | **1.2068** | **96** | **4.8892** | **0.0011** |
| **Artist F. verticillioides 12-6-12 - Artist No treatment No treatment** | **4.8** | **1.2068** | **96** | **3.9776** | **0.0350** |
| **Artist No treatment 12-6-12 - Euforia Consortium No treatment** | **4.9** | **1.2068** | **96** | **4.0605** | **0.0261** |
| **Artist No treatment 12-6-12 - Euforia F. graminearum No treatment** | **4.9** | **1.2068** | **96** | **4.0605** | **0.0261** |
| **Euforia Consortium No treatment - Euforia No treatment No treatment** | **-6.3** | **1.2068** | **96** | **-5.2206** | **2.8e-04** |
| **Euforia F. graminearum No treatment - Euforia No treatment No treatment** | **-6.3** | **1.2068** | **96** | **-5.2206** | **2.8e-04** |
| **Artist F. verticillioides No treatment - Euforia No treatment No treatment** | **-4.8** | **1.2068** | **96** | **-3.9776** | **0.0350** |
| **Artist No treatment No treatment - Euforia No treatment No treatment** | **-5.2** | **1.2068** | **96** | **-4.3091** | **0.0104** |
| *Holm correction. Bold: p < α.* | | | | | |

## Soil trials [Root]

| **Comparison** | **Estimate** | **SE** | **df** | **t** | **p_adj** |
| --- | --- | --- | --- | --- | --- |
| **Artist F. graminearum 12-6-12 - Artist Consortium 12-O-12** | **3.4** | **0.8016** | **96** | **4.2417** | **0.0138** |
| **Artist F. graminearum 12-6-12 - Euforia Consortium 12-O-12** | **3.2** | **0.8016** | **96** | **3.9922** | **0.0342** |
| **Artist F. graminearum 12-6-12 - Euforia F. graminearum No treatment** | **4.3** | **0.8016** | **96** | **5.3645** | **1.5e-04** |
| **Artist F. graminearum 12-6-12 - Artist F. verticillioides No treatment** | **3.3** | **0.8016** | **96** | **4.1170** | **0.0219** |
| **Artist F. graminearum 12-6-12 - Euforia F. verticillioides No treatment** | **3.8** | **0.8016** | **96** | **4.7407** | **0.0020** |
| **Artist F. graminearum 12-6-12 - Euforia No treatment No treatment** | **4.5** | **0.8016** | **96** | **5.6140** | **5.3e-05** |
| **Artist Consortium No treatment - Euforia F. graminearum No treatment** | **3.3** | **0.8016** | **96** | **4.1170** | **0.0219** |
| **Artist Consortium No treatment - Euforia No treatment No treatment** | **3.5** | **0.8016** | **96** | **4.3665** | **0.0086** |
| **Euforia F. graminearum No treatment - Artist No treatment No treatment** | **-3.6** | **0.8016** | **96** | **-4.4912** | **0.0054** |
| **Artist No treatment No treatment - Euforia No treatment No treatment** | **3.8** | **0.8016** | **96** | **4.7407** | **0.0020** |
| *Holm correction. Bold: p < α.* | | | | | |

## Soil trials [Whole plant]

| **Factor** | **Comparison** | **Estimate** | **SE** | **df** | **t** | **p_adj** |
| --- | --- | --- | --- | --- | --- | --- |
| **Variety** | **Artist - Euforia** | **-54.200** | **4.3380** | **96** | **-12.4942** | **7.7e-22** |
| **Treatment** | **12-6-12 - 12-O-12** | **33.275** | **6.6412** | **96** | **5.0104** | **4.9e-06** |
| **Treatment** | **12-6-12 - No treatment** | **53.425** | **6.6412** | **96** | **8.0444** | **6.9e-12** |
| **Treatment** | **12-O-12 - No treatment** | **20.150** | **6.6412** | **96** | **3.0341** | **0.0031** |
| *Holm correction. Bold: p < α.* | | | | | | |

# Descriptive Statistics

*Mean (M), standard deviation (SD), and median (Mdn) per treatment combination.*

## Soil trials [Above-ground]

| **Variety** | **Pathogen** | **Treatment** | **n** | **M** | **SD** | **Mdn** |
| --- | --- | --- | --- | --- | --- | --- |
| Artist | Consortium | 12-6-12 | 5 | 17.3 | 0.57 | 17.5 |
| Artist | Consortium | 12-O-12 | 5 | 14.7 | 3.17 | 16.5 |
| Artist | Consortium | No treatment | 5 | 16.2 | 0.97 | 16.5 |
| Artist | F. graminearum | 12-6-12 | 5 | 19.3 | 0.76 | 19.5 |
| Artist | F. graminearum | 12-O-12 | 5 | 16.0 | 1.06 | 16.0 |
| Artist | F. graminearum | No treatment | 5 | 15.8 | 0.91 | 16.0 |
| Artist | F. verticillioides | 12-6-12 | 5 | 17.8 | 0.76 | 17.5 |
| Artist | F. verticillioides | 12-O-12 | 5 | 16.3 | 1.15 | 16.5 |
| Artist | F. verticillioides | No treatment | 5 | 13.4 | 1.60 | 13.0 |
| Artist | No treatment | 12-6-12 | 5 | 16.8 | 1.48 | 17.5 |
| Artist | No treatment | 12-O-12 | 5 | 16.0 | 1.46 | 16.5 |
| Artist | No treatment | No treatment | 5 | 13.0 | 2.85 | 12.5 |
| Euforia | Consortium | 12-6-12 | 5 | 18.2 | 1.25 | 18.0 |
| Euforia | Consortium | 12-O-12 | 5 | 15.0 | 1.80 | 15.5 |
| Euforia | Consortium | No treatment | 5 | 11.9 | 3.75 | 12.0 |
| Euforia | F. graminearum | 12-6-12 | 5 | 16.5 | 0.87 | 16.0 |
| Euforia | F. graminearum | 12-O-12 | 5 | 15.6 | 2.46 | 16.0 |
| Euforia | F. graminearum | No treatment | 5 | 11.9 | 2.70 | 12.0 |
| Euforia | F. verticillioides | 12-6-12 | 5 | 14.9 | 1.29 | 15.0 |
| Euforia | F. verticillioides | 12-O-12 | 5 | 13.8 | 3.15 | 14.5 |
| Euforia | F. verticillioides | No treatment | 5 | 16.4 | 1.39 | 17.0 |
| Euforia | No treatment | 12-6-12 | 5 | 15.6 | 1.64 | 15.5 |
| Euforia | No treatment | 12-O-12 | 5 | 15.3 | 1.68 | 15.0 |
| Euforia | No treatment | No treatment | 5 | 18.2 | 2.14 | 19.0 |
| *M = mean; SD = standard deviation; Mdn = median.* | | | | | | |

## Soil trials [Root]

| **Variety** | **Pathogen** | **Treatment** | **n** | **M** | **SD** | **Mdn** |
| --- | --- | --- | --- | --- | --- | --- |
| Artist | Consortium | 12-6-12 | 5 | 6.6 | 1.29 | 6.0 |
| Artist | Consortium | 12-O-12 | 5 | 4.8 | 1.44 | 5.5 |
| Artist | Consortium | No treatment | 5 | 7.2 | 2.02 | 7.0 |
| Artist | F. graminearum | 12-6-12 | 5 | 8.2 | 1.35 | 8.0 |
| Artist | F. graminearum | 12-O-12 | 5 | 5.6 | 0.65 | 5.5 |
| Artist | F. graminearum | No treatment | 5 | 6.1 | 0.96 | 6.5 |
| Artist | F. verticillioides | 12-6-12 | 5 | 6.8 | 0.76 | 6.5 |
| Artist | F. verticillioides | 12-O-12 | 5 | 6.7 | 0.97 | 7.0 |
| Artist | F. verticillioides | No treatment | 5 | 4.9 | 0.65 | 5.0 |
| Artist | No treatment | 12-6-12 | 5 | 6.4 | 0.65 | 6.0 |
| Artist | No treatment | 12-O-12 | 5 | 5.5 | 2.09 | 5.0 |
| Artist | No treatment | No treatment | 5 | 7.5 | 2.06 | 9.0 |
| Euforia | Consortium | 12-6-12 | 5 | 5.3 | 0.97 | 5.0 |
| Euforia | Consortium | 12-O-12 | 5 | 5.0 | 1.00 | 4.5 |
| Euforia | Consortium | No treatment | 5 | 5.4 | 1.29 | 5.0 |
| Euforia | F. graminearum | 12-6-12 | 5 | 5.6 | 0.65 | 5.5 |
| Euforia | F. graminearum | 12-O-12 | 5 | 5.4 | 1.52 | 6.0 |
| Euforia | F. graminearum | No treatment | 5 | 3.9 | 1.25 | 4.0 |
| Euforia | F. verticillioides | 12-6-12 | 5 | 5.3 | 0.97 | 5.0 |
| Euforia | F. verticillioides | 12-O-12 | 5 | 5.7 | 1.60 | 5.5 |
| Euforia | F. verticillioides | No treatment | 5 | 4.4 | 0.65 | 4.5 |
| Euforia | No treatment | 12-6-12 | 5 | 6.8 | 1.64 | 6.5 |
| Euforia | No treatment | 12-O-12 | 5 | 5.1 | 1.19 | 5.0 |
| Euforia | No treatment | No treatment | 5 | 3.7 | 0.76 | 3.5 |
| *M = mean; SD = standard deviation; Mdn = median.* | | | | | | |

## Soil trials [Whole plant]

| **Variety** | **Pathogen** | **Treatment** | **n** | **M** | **SD** | **Mdn** |
| --- | --- | --- | --- | --- | --- | --- |
| Artist | Consortium | 12-6-12 | 5 | 17.3 | 0.57 | 17.5 |
| Artist | Consortium | 12-O-12 | 5 | 14.7 | 3.17 | 16.5 |
| Artist | Consortium | No treatment | 5 | 16.2 | 0.97 | 16.5 |
| Artist | F. graminearum | 12-6-12 | 5 | 19.3 | 0.76 | 19.5 |
| Artist | F. graminearum | 12-O-12 | 5 | 16.0 | 1.06 | 16.0 |
| Artist | F. graminearum | No treatment | 5 | 15.8 | 0.91 | 16.0 |
| Artist | F. verticillioides | 12-6-12 | 5 | 17.8 | 0.76 | 17.5 |
| Artist | F. verticillioides | 12-O-12 | 5 | 16.3 | 1.15 | 16.5 |
| Artist | F. verticillioides | No treatment | 5 | 13.4 | 1.60 | 13.0 |
| Artist | No treatment | 12-6-12 | 5 | 16.8 | 1.48 | 17.5 |
| Artist | No treatment | 12-O-12 | 5 | 16.0 | 1.46 | 16.5 |
| Artist | No treatment | No treatment | 5 | 13.0 | 2.85 | 12.5 |
| Euforia | Consortium | 12-6-12 | 5 | 23.5 | 0.87 | 23.0 |
| Euforia | Consortium | 12-O-12 | 5 | 20.0 | 2.42 | 19.5 |
| Euforia | Consortium | No treatment | 5 | 17.3 | 4.48 | 16.0 |
| Euforia | F. graminearum | 12-6-12 | 5 | 22.1 | 0.74 | 22.0 |
| Euforia | F. graminearum | 12-O-12 | 5 | 21.0 | 3.04 | 21.0 |
| Euforia | F. graminearum | No treatment | 5 | 15.8 | 1.52 | 15.5 |
| Euforia | F. verticillioides | 12-6-12 | 5 | 20.2 | 1.20 | 19.5 |
| Euforia | F. verticillioides | 12-O-12 | 5 | 19.5 | 4.44 | 20.0 |
| Euforia | F. verticillioides | No treatment | 5 | 20.8 | 1.72 | 21.5 |
| Euforia | No treatment | 12-6-12 | 5 | 22.4 | 2.04 | 23.0 |
| Euforia | No treatment | 12-O-12 | 5 | 20.4 | 1.52 | 20.5 |
| Euforia | No treatment | No treatment | 5 | 21.9 | 2.33 | 23.0 |
| *M = mean; SD = standard deviation; Mdn = median.* | | | | | | |

# Interaction Plots

*Cell means plotted by factor level. Parallel lines indicate no interaction; crossing or diverging lines indicate a significant interaction.*

## Soil trials [Above-ground]


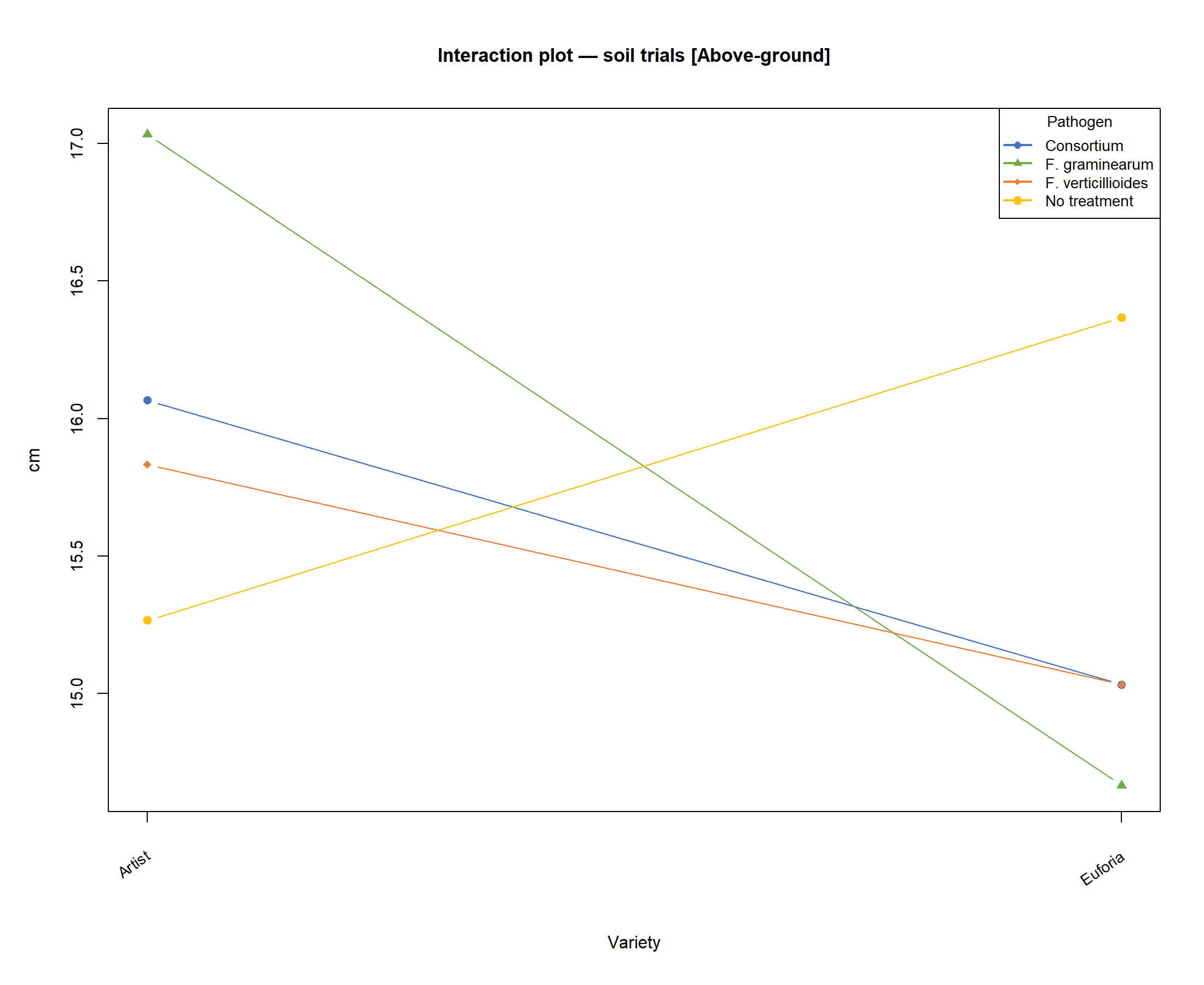


## Soil trials [Root]


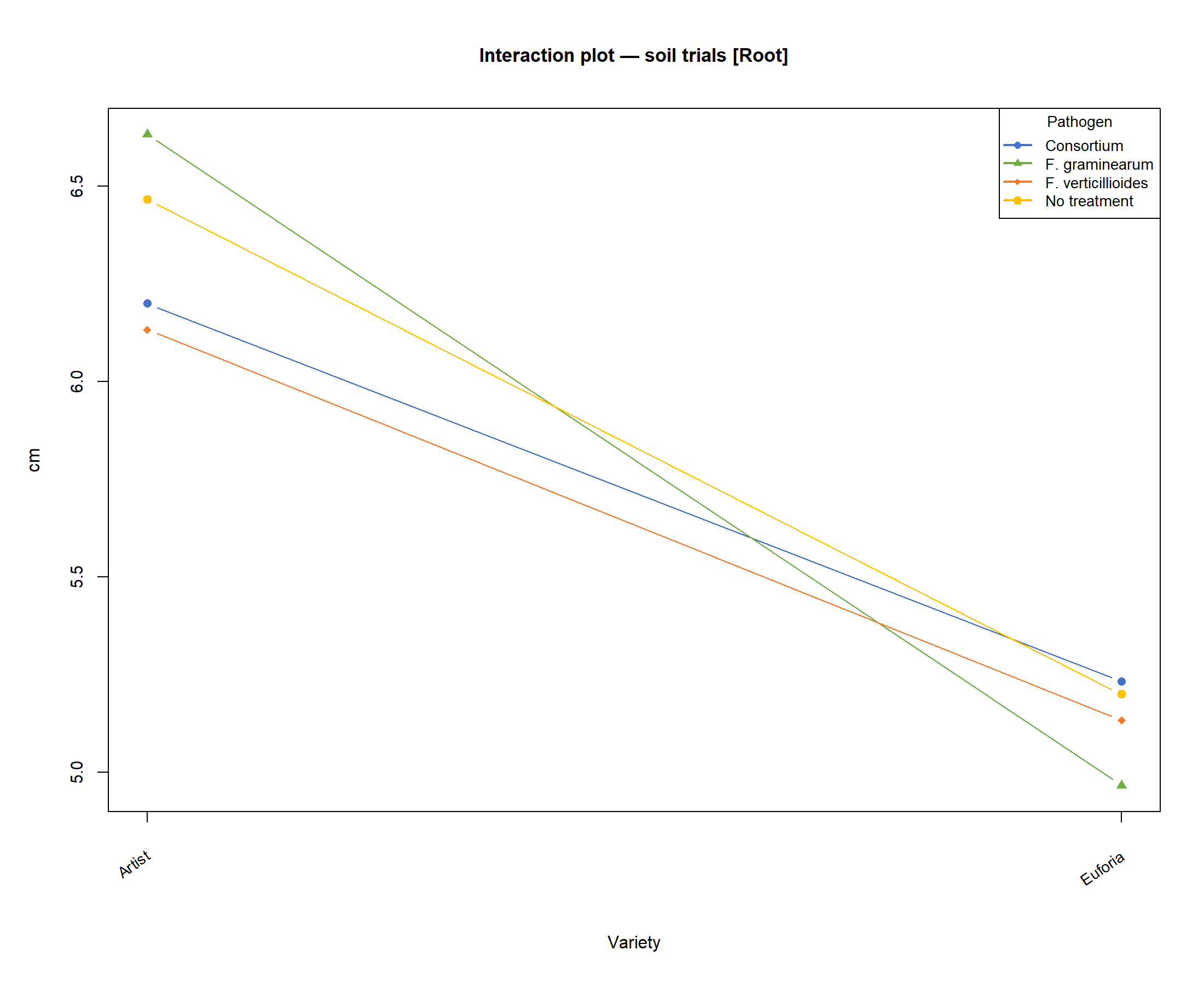


## Soil trials [Whole plant]


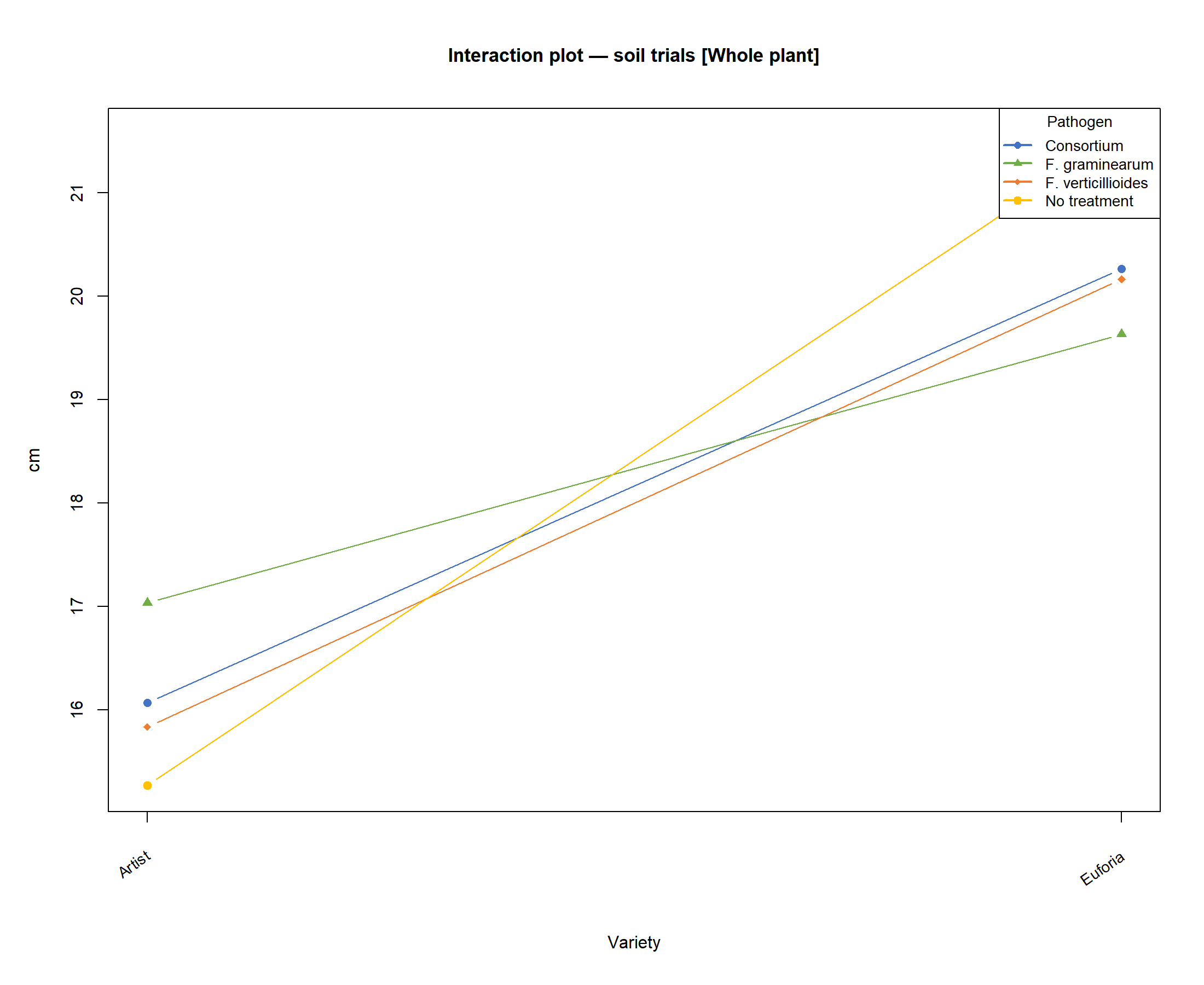


# References

1. Fox, J.; Weisberg, S. *An R Companion to Applied Regression*, 3rd ed.; Sage: Thousand Oaks, CA, USA, 2019. Available online: https://us.sagepub.com/en-us/nam/an-r-companion-to-applied-regression/book246125 (accessed on 28 April 2026).
2. Lenth, R.V. *emmeans: Estimated Marginal Means, Aka Least-Squares Means*, R Package Version 1.10.0; R Foundation: Vienna, Austria, 2024. Available online: https://CRAN.R-project.org/package=emmeans (accessed on 28 April 2026).
3. Kay, M.; Wobbrock, J.O. *ARTool: Aligned Rank Transform for Nonparametric Factorial ANOVAs*, R Package Version 0.11.1; R Foundation: Vienna, Austria, 2021. Available online: https://CRAN.R-project.org/package=ARTool (accessed on 28 April 2026).
