# Supplementary material for: Preliminary Evaluation of Gemini-Surfactant-Based Formulations for Antifungal Seed Treatment in Wheat
Source: Molecules. 2026 May 8;31(10):1568. doi: 10.3390/molecules31101568 (PMC13209164; doi:10.3390/molecules31101568)
Supplement: Supplementary file 1 [file molecules-31-01568-s001.zip › molecules-4247638-supplementary/supplementary_material_I_synthetic_medium_trials.docx]

**Statistical Supplementary Material: Synthetic medium trials**

*• Correction: Holm • α = 0.05*

# Methods

All statistical analyses were performed in R (v4.5.2; R Core Team, 2025). Experimental design was detected automatically from CSV column structure (n = 3 per group), and confirmed manually. Normality of model residuals was assessed with the Shapiro-Wilk test; variance homogeneity with Levene's test (Fox & Weisberg, 2019). Where normality was violated, five transformations were evaluated (log, square-root, arcsine, Box-Cox, reciprocal; Venables & Ripley, 2002) and the transformation producing the greatest improvement in Shapiro-Wilk p-value was selected. Where factorial residuals could not be normalised, Aligned Rank Transform ANOVA (ART-ANOVA) was applied (Wobbrock et al., 2011) with pairwise ART contrasts (Holm correction). Statistical significance was set at α = 0.05.

# Results Summary

## Synthetic medium (MEA)

Factorial ANOVA revealed significant effects of: Variety (F(1,120) =  67.2527, p = 3.0e-13, partial η² = —); Pathogen (F(2,120) = 579.8723, p = 2.1e-62, partial η² = —); Sterility (F(1,120) = 203.4013, p = 1.3e-27, partial η² = —); Treatment (F(4,120) = 137.6348, p = 7.4e-44, partial η² = —); Variety:Pathogen (F(2,120) =  71.2954, p = 3.9e-21, partial η² = —); Variety:Sterility (F(1,120) =  52.7041, p = 4.2e-11, partial η² = —); Pathogen:Sterility (F(2,120) =  96.8993, p = 8.9e-26, partial η² = —); Variety:Treatment (F(4,120) =  34.7789, p = 2.9e-19, partial η² = —); Pathogen:Treatment (F(8,120) = 155.0294, p = 1.6e-59, partial η² = —); Sterility:Treatment (F(4,120) =  52.7870, p = 1.4e-25, partial η² = —); Variety:Pathogen:Sterility (F(2,120) =  60.0235, p = 8.6e-19, partial η² = —); Variety:Pathogen:Treatment (F(8,120) =  25.8518, p = 8.1e-23, partial η² = —); Variety:Sterility:Treatment (F(4,120) =  49.1823, p = 2.0e-24, partial η² = —); Pathogen:Sterility:Treatment (F(8,120) =  29.0509, p = 9.9e-25, partial η² = —); Variety:Pathogen:Sterility:Treatment (F(8,120) =  60.5463, p = 1.5e-38, partial η² = —). Non-significant terms are omitted.

# Abbreviations

| **Abbreviation** | **Definition** |
| --- | --- |
| ANOVA | Analysis of Variance |
| ART | Aligned Rank Transform (non-parametric factorial; Wobbrock et al., 2011) |
| W | Shapiro-Wilk W statistic |
| F | F-ratio |
| p | p-value |
| α | Significance level (α = 0.05) |
| df | Degrees of freedom |
| M | Mean |
| SD | Standard deviation |
| Mdn | Median |
| HSD | Tukey Honestly Significant Difference |
| BH | Benjamini-Hochberg false discovery rate |
| η² | Eta-squared: proportion of total variance explained |
| partialη² | Partial eta-squared: variance explained, partialling out other terms |
| ω² | Omega-squared: bias-corrected eta-squared |
| partialω² | Partial omega-squared: bias-corrected partial omega-squared |

# Assumptions

## Synthetic medium (MEA)

| **Test** | **Statistic** | **p** | **Conclusion** |
| --- | --- | --- | --- |
| Shapiro-Wilk (residuals) | 0.7499 | 3.4e-16 | Non-normal |
| Levene's test | 1.0460 | 0.4108 | Equal |

# Main Statistical Tests

## Synthetic medium (MEA)

| **Term** | **Df** | **df_res** | **F** | **p** |
| --- | --- | --- | --- | --- |
| **Variety** | **1** | **120** | **67.2527** | **3.0e-13** |
| **Pathogen** | **2** | **120** | **579.8723** | **2.1e-62** |
| **Sterility** | **1** | **120** | **203.4013** | **1.3e-27** |
| **Treatment** | **4** | **120** | **137.6348** | **7.4e-44** |
| **Variety:Pathogen** | **2** | **120** | **71.2954** | **3.9e-21** |
| **Variety:Sterility** | **1** | **120** | **52.7041** | **4.2e-11** |
| **Pathogen:Sterility** | **2** | **120** | **96.8993** | **8.9e-26** |
| **Variety:Treatment** | **4** | **120** | **34.7789** | **2.9e-19** |
| **Pathogen:Treatment** | **8** | **120** | **155.0294** | **1.6e-59** |
| **Sterility:Treatment** | **4** | **120** | **52.7870** | **1.4e-25** |
| **Variety:Pathogen:Sterility** | **2** | **120** | **60.0235** | **8.6e-19** |
| **Variety:Pathogen:Treatment** | **8** | **120** | **25.8518** | **8.1e-23** |
| **Variety:Sterility:Treatment** | **4** | **120** | **49.1823** | **2.0e-24** |
| **Pathogen:Sterility:Treatment** | **8** | **120** | **29.0509** | **9.9e-25** |
| **Variety:Pathogen:Sterility:Treatment** | **8** | **120** | **60.5463** | **1.5e-38** |
| *Bold: p < α.* | | | | |

# Post-hoc Comparisons (significant at p < 0.05)

*Only comparisons with p < 0.05 shown.*

## Synthetic medium (MEA)

| **Factor** | **Comparison** | **Estimate** | **SE** | **df** | **t** | **p_adj** |
| --- | --- | --- | --- | --- | --- | --- |
| **Variety** | **Artist - Euforia** | **59.8000** | **7.2920** | **120** | **8.2008** | **3.0e-13** |
| **Pathogen** | **Consortium - Monoculture** | **-60.0000** | **3.5237** | **120** | **-17.0275** | **1.7e-33** |
| **Pathogen** | **Consortium - No pathogen** | **60.0000** | **3.5237** | **120** | **17.0275** | **1.7e-33** |
| **Pathogen** | **Monoculture - No pathogen** | **120.0000** | **3.5237** | **120** | **34.0550** | **4.8e-63** |
| **Sterility** | **Non-sterile - Sterile** | **-81.0222** | **5.6810** | **120** | **-14.2619** | **1.3e-27** |
| **Treatment** | **12-6-12/2S - 12-6-12/S** | **23.5139** | **6.2610** | **120** | **3.7556** | **2.7e-04** |
| **Treatment** | **12-6-12/2S - 12-O-12/2S** | **-63.5694** | **6.2610** | **120** | **-10.1532** | **4.4e-17** |
| **Treatment** | **12-6-12/2S - 12-O-12/S** | **-31.6667** | **6.2610** | **120** | **-5.0577** | **3.9e-06** |
| **Treatment** | **12-6-12/2S - No treatment** | **-107.9306** | **6.2610** | **120** | **-17.2384** | **2.6e-33** |
| **Treatment** | **12-6-12/S - 12-O-12/2S** | **-87.0833** | **6.2610** | **120** | **-13.9088** | **7.0e-26** |
| **Treatment** | **12-6-12/S - 12-O-12/S** | **-55.1806** | **6.2610** | **120** | **-8.8133** | **5.6e-14** |
| **Treatment** | **12-6-12/S - No treatment** | **-131.4444** | **6.2610** | **120** | **-20.9940** | **5.5e-41** |
| **Treatment** | **12-O-12/2S - 12-O-12/S** | **31.9028** | **6.2610** | **120** | **5.0954** | **3.9e-06** |
| **Treatment** | **12-O-12/2S - No treatment** | **-44.3611** | **6.2610** | **120** | **-7.0853** | **4.1e-10** |
| **Treatment** | **12-O-12/S - No treatment** | **-76.2639** | **6.2610** | **120** | **-12.1807** | **7.2e-22** |
| *Holm correction. Bold: p < α.* | | | | | | |

# Descriptive Statistics

*Mean (M), standard deviation (SD), and median (Mdn) per treatment combination.*

## Synthetic medium (MEA)

| **Variety** | **Pathogen** | **Sterility** | **Treatment** | **n** | **M** | **SD** | **Mdn** |
| --- | --- | --- | --- | --- | --- | --- | --- |
| Artist | Consortium | Non-sterile | 12-6-12/2S | 3 | 5.00 | 1.00 | 5 |
| Artist | Consortium | Non-sterile | 12-6-12/S | 3 | 7.00 | 0.00 | 7 |
| Artist | Consortium | Non-sterile | 12-O-12/2S | 3 | 4.00 | 0.00 | 4 |
| Artist | Consortium | Non-sterile | 12-O-12/S | 3 | 9.00 | 0.00 | 9 |
| Artist | Consortium | Non-sterile | No treatment | 3 | 100.00 | 0.00 | 100 |
| Artist | Consortium | Sterile | 12-6-12/2S | 3 | 24.33 | 0.58 | 24 |
| Artist | Consortium | Sterile | 12-6-12/S | 3 | 19.00 | 1.73 | 20 |
| Artist | Consortium | Sterile | 12-O-12/2S | 3 | 10.00 | 1.00 | 10 |
| Artist | Consortium | Sterile | 12-O-12/S | 3 | 42.00 | 1.73 | 43 |
| Artist | Consortium | Sterile | No treatment | 3 | 100.00 | 0.00 | 100 |
| Artist | Monoculture | Non-sterile | 12-6-12/2S | 3 | 48.00 | 8.19 | 46 |
| Artist | Monoculture | Non-sterile | 12-6-12/S | 3 | 33.00 | 3.46 | 35 |
| Artist | Monoculture | Non-sterile | 12-O-12/2S | 3 | 58.00 | 8.66 | 53 |
| Artist | Monoculture | Non-sterile | 12-O-12/S | 3 | 45.00 | 1.00 | 45 |
| Artist | Monoculture | Non-sterile | No treatment | 3 | 100.00 | 0.00 | 100 |
| Artist | Monoculture | Sterile | 12-6-12/2S | 3 | 52.00 | 9.54 | 57 |
| Artist | Monoculture | Sterile | 12-6-12/S | 3 | 45.33 | 2.08 | 46 |
| Artist | Monoculture | Sterile | 12-O-12/2S | 3 | 95.00 | 4.81 | 99 |
| Artist | Monoculture | Sterile | 12-O-12/S | 3 | 49.00 | 4.58 | 48 |
| Artist | Monoculture | Sterile | No treatment | 3 | 100.00 | 0.00 | 100 |
| Artist | No pathogen | Non-sterile | 12-6-12/2S | 3 | 2.67 | 0.58 | 3 |
| Artist | No pathogen | Non-sterile | 12-6-12/S | 3 | 0.67 | 0.58 | 1 |
| Artist | No pathogen | Non-sterile | 12-O-12/2S | 3 | 2.00 | 1.00 | 2 |
| Artist | No pathogen | Non-sterile | 12-O-12/S | 3 | 1.00 | 0.00 | 1 |
| Artist | No pathogen | Non-sterile | No treatment | 3 | 90.00 | 0.00 | 90 |
| Artist | No pathogen | Sterile | 12-6-12/2S | 3 | 1.33 | 1.15 | 2 |
| Artist | No pathogen | Sterile | 12-6-12/S | 3 | 1.00 | 0.00 | 1 |
| Artist | No pathogen | Sterile | 12-O-12/2S | 3 | 1.67 | 0.58 | 2 |
| Artist | No pathogen | Sterile | 12-O-12/S | 3 | 1.33 | 0.58 | 1 |
| Artist | No pathogen | Sterile | No treatment | 3 | 95.00 | 0.00 | 95 |
| Euforia | Consortium | Non-sterile | 12-6-12/2S | 3 | 2.00 | 0.00 | 2 |
| Euforia | Consortium | Non-sterile | 12-6-12/S | 3 | 12.00 | 1.00 | 12 |
| Euforia | Consortium | Non-sterile | 12-O-12/2S | 3 | 4.00 | 0.00 | 4 |
| Euforia | Consortium | Non-sterile | 12-O-12/S | 3 | 3.00 | 0.00 | 3 |
| Euforia | Consortium | Non-sterile | No treatment | 3 | 100.00 | 0.00 | 100 |
| Euforia | Consortium | Sterile | 12-6-12/2S | 3 | 5.00 | 0.00 | 5 |
| Euforia | Consortium | Sterile | 12-6-12/S | 3 | 10.00 | 0.00 | 10 |
| Euforia | Consortium | Sterile | 12-O-12/2S | 3 | 4.00 | 0.00 | 4 |
| Euforia | Consortium | Sterile | 12-O-12/S | 3 | 19.33 | 1.53 | 19 |
| Euforia | Consortium | Sterile | No treatment | 3 | 100.00 | 0.00 | 100 |
| Euforia | Monoculture | Non-sterile | 12-6-12/2S | 3 | 37.00 | 1.73 | 38 |
| Euforia | Monoculture | Non-sterile | 12-6-12/S | 3 | 44.00 | 1.73 | 45 |
| Euforia | Monoculture | Non-sterile | 12-O-12/2S | 3 | 77.00 | 2.00 | 77 |
| Euforia | Monoculture | Non-sterile | 12-O-12/S | 3 | 34.33 | 7.51 | 30 |
| Euforia | Monoculture | Non-sterile | No treatment | 3 | 100.00 | 0.00 | 100 |
| Euforia | Monoculture | Sterile | 12-6-12/2S | 3 | 63.33 | 3.06 | 64 |
| Euforia | Monoculture | Sterile | 12-6-12/S | 3 | 53.00 | 1.73 | 52 |
| Euforia | Monoculture | Sterile | 12-O-12/2S | 3 | 58.00 | 2.00 | 58 |
| Euforia | Monoculture | Sterile | 12-O-12/S | 3 | 68.00 | 4.58 | 67 |
| Euforia | Monoculture | Sterile | No treatment | 3 | 100.00 | 0.00 | 100 |
| Euforia | No pathogen | Non-sterile | 12-6-12/2S | 3 | 0.00 | 0.00 | 0 |
| Euforia | No pathogen | Non-sterile | 12-6-12/S | 3 | 0.33 | 0.58 | 0 |
| Euforia | No pathogen | Non-sterile | 12-O-12/2S | 3 | 1.67 | 0.58 | 2 |
| Euforia | No pathogen | Non-sterile | 12-O-12/S | 3 | 0.67 | 1.15 | 0 |
| Euforia | No pathogen | Non-sterile | No treatment | 3 | 85.00 | 5.00 | 85 |
| Euforia | No pathogen | Sterile | 12-6-12/2S | 3 | 1.33 | 0.58 | 1 |
| Euforia | No pathogen | Sterile | 12-6-12/S | 3 | 1.33 | 0.58 | 1 |
| Euforia | No pathogen | Sterile | 12-O-12/2S | 3 | 1.33 | 0.58 | 1 |
| Euforia | No pathogen | Sterile | 12-O-12/S | 3 | 1.33 | 0.58 | 1 |
| Euforia | No pathogen | Sterile | No treatment | 3 | 100.00 | 0.00 | 100 |
| *M = mean; SD = standard deviation; Mdn = median.* | | | | | | | |

# Interaction Plots

*Cell means plotted by factor level. Parallel lines indicate no interaction; crossing or diverging lines indicate a significant interaction (see ANOVA table).*

## Synthetic medium (MEA)


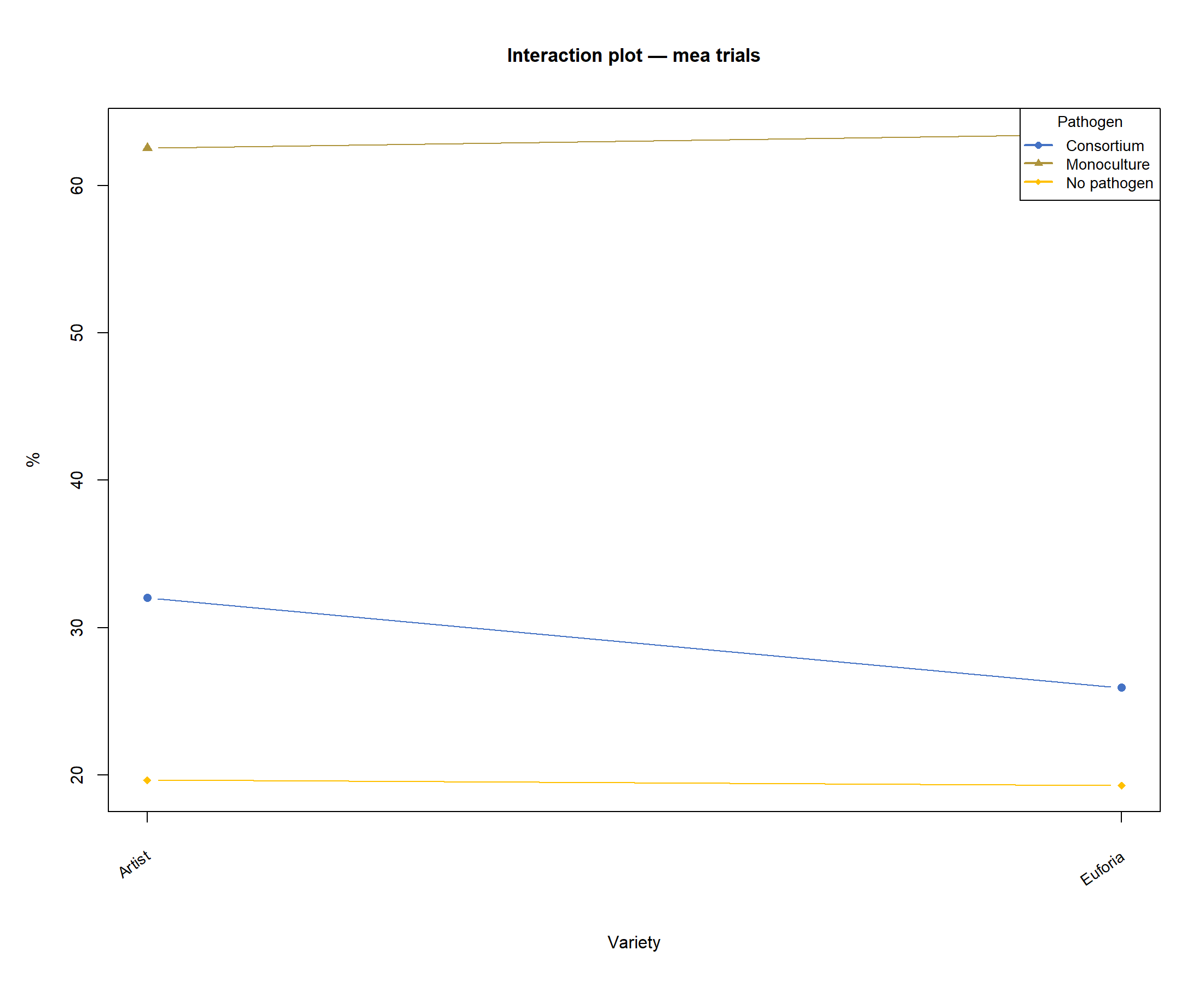


# References

1. Fox, J.; Weisberg, S. *An R Companion to Applied Regression*, 3rd ed.; Sage: Thousand Oaks, CA, USA, 2019. Available online: https://us.sagepub.com/en-us/nam/an-r-companion-to-applied-regression/book246125 (accessed on 28 April 2026).
2. Lenth, R.V. *emmeans: Estimated Marginal Means, Aka Least-Squares Means*, R Package Version 1.10.0; R Foundation: Vienna, Austria, 2024. Available online: https://CRAN.R-project.org/package=emmeans (accessed on 28 April 2026).
3. Kay, M.; Wobbrock, J.O. *ARTool: Aligned Rank Transform for Nonparametric Factorial ANOVAs*, R Package Version 0.11.1; R Foundation: Vienna, Austria, 2021. Available online: https://CRAN.R-project.org/package=ARTool (accessed on 28 April 2026).
